# Effects of Elevated CO_2_ on Yield and Nutritional Quality of Kale and Spinach: A Meta-Analysis

**DOI:** 10.3390/biology15020152

**Published:** 2026-01-15

**Authors:** Jiata U. Ekele, Joseph O. Obaje, Susanne R. K. Zajitschek, Richard J. Webster, Fatima Perez de Heredia, Katie E. Lane, Abdulmannan Fadel, Rachael C. Symonds

**Affiliations:** 1 School of Biological and Environmental Sciences, Liverpool John Moores University, Byrom Street, Liverpool L3 3AF, UKs.r.zajitschek@ljmu.ac.uk (S.R.K.Z.); r.c.symonds@ljmu.ac.uk (R.C.S.); 2 Institute of Health Research, Liverpool John Moores University, Byrom Street, Liverpool L3 3AF, UK; 3 Liverpool Centre for Cardiovascular Science, Liverpool L7 8TX, UK; 4 Department of Sports and Exercise Sciences, Liverpool John Moores University, Byrom Street, Liverpool L3 3AF, UK; 5 Department of Nutrition and Health, College of Medicine and Health Sciences, United Arab Emirates University, Al Ain P.O. Box 1555, United Arab Emirates

**Keywords:** climate change, crop nutrients, eCO_2_, elevated carbon dioxide, food security, kale, nutrition, spinach

## Abstract

Elevated atmospheric CO_2_ is known to alter plant physiology, yet its specific effects on nutrient-rich leafy vegetables remain insufficiently quantified. This study aimed to examine how eCO_2_ influences yield and nutritional quality in kale (*Brassica oleracea*) and spinach (*Spinacia oleracea*) through the first meta-analysis focused exclusively on these crops. Following the Collaboration for Environmental Evidence (CEE) guidelines, we systematically reviewed eligible studies and conducted a random-effects meta-analysis to evaluate overall and subgroup responses based on CO_2_ concentration, crop type and exposure duration. Effect sizes were calculated using Hedges’ g with 95% confidence intervals. The analysis showed that eCO_2_ significantly increased biomass in spinach (g = 1.21) and kale (g = 0.97). However, protein content declined in both crops (spinach: g = −0.76; kale: g = −0.61), and mineral concentrations, particularly calcium and magnesium, were reduced, with spinach exhibiting stronger nutrient losses overall. The variability in response across different CO_2_ concentrations and exposure times further underscores the complexity of eCO_2_ effects. These results highlight a trade-off between productivity and nutritional quality under future CO_2_ conditions. Addressing this challenge will require strategies such as targeted breeding programmes, biofortification, precision agriculture and improved sustainable agricultural practices to maintain nutrient density. This research provides critical evidence for policymakers and scientists to design sustainable food systems that safeguard public health in a changing climate.

## Simple Summary

Rising carbon dioxide levels in the atmosphere are changing how crops grow, with implications for food supply and nutrition. This study is the first meta-analysis focused specifically on kale and spinach, combining data from 13 studies and 339 effect sizes to understand how elevated CO_2_ (at different ranges from 650 ppm to above 3000 ppm) affects yield and nutritional quality. We found that higher CO_2_ generally boosts growth and yield (Hedges’ g ≈ 1.04) but reduces important nutrients: protein declined in both crops (spinach: g = −0.76; kale: g = −0.61), and minerals such as calcium and magnesium fell sharply in spinach. Spinach responded with greater growth than kale but also showed more nutrient loss. These changes could have implications for many populations who rely on these vegetables for essential vitamins and minerals. If future trends continue, nutrient dilution could lead to health problems like deficiencies and related diseases. These findings underscore the need for targeted farming strategies and plant breeding programmes to preserve nutritional quality while meeting food demand in a changing climate. This research provides evidence to guide policymakers, farmers, and scientists in planning sustainable food systems that protect public health.

## 1. Introduction

Climate change is exerting unprecedented pressure on global agricultural systems, and rising atmospheric CO_2_ is a key driver of this change [1]. Understanding its impact on crop production and nutritional quality is critical for ensuring food security, sustainable diet and human health [2]. While cereals and legumes have been widely studied, leafy vegetables such as kale (*Brassica oleracea*) and spinach (*Spinacia oleracea*) remain underexplored despite their global nutritional importance [3,4,5]. These crops are rich sources of vitamins, minerals, and phytochemicals, making them essential for healthy diets worldwide.

Kale contains high concentrations of vitamins A, C, and K, along with glucosinolates and minerals important for bone health [6,7]. Spinach is similarly nutrient-dense, providing vitamins A, C, and K, as well as essential minerals such as iron, magnesium, and folate [8,9]. Their consumption has increased globally, and they form a significant part of both plant-based and mixed diets [10,11,12]. Given their micronutrient value, any reductions in these nutrient levels due to eCO_2_-induced nutrient dilution could have significant public health implications, especially among populations that rely heavily on greens for their micronutrient intake [13,14].

Preliminary research suggests that eCO_2_ may differentially impact various nutritional components in food crops potentially increasing carbohydrate content but decreasing protein and mineral concentrations [15]. Long-term Free-Air CO_2_ Enrichment (FACE) and controlled-environment studies indicate that crop responses to eCO_2_ vary depending on the CO_2_ concentration, duration of exposure and crop variety [16,17,18]. However, very few studies have focused specifically on leafy vegetables such as kale and spinach.

Despite the well-documented nutritional interest of kale and spinach, the effects of eCO_2_ on their constituents have been less studied compared to staple crops. This lack of consolidated evidence limits our understanding of how future atmospheric CO_2_ conditions may alter the nutritional quality of these widely consumed leafy greens.

To address this gap, this study presents the first meta-analysis that statistically combines results from multiple studies on kale and spinach to provide more robust and precise conclusions than any single study can offer. By synthesising 13 studies and 339 effect sizes using random-effects models, we evaluate how variations in CO_2_ concentration, crop type, and exposure duration influence yield and nutritional quality, with the aim of informing strategies to mitigate adverse nutritional effects under future climate scenarios.

## 2. Materials and Methods

Our meta-analysis adhered to a systematic approach, following the standards established by the Collaboration for Environmental Evidence and Standards for Evidence Synthesis [19]. Our construction was based on a systematic map (Supplement Material, pp. 1–3) which provided the basis for our research questions and study selection criteria, detailing the effects of eCO_2_ on various crops, including kale and spinach. Our main research question was: “What published evidence exists for the effects of eCO_2_ on the nutritional components of kale and spinach crops?” Our analysis expanded on this question by incorporating secondary questions related to differences in effects by crop type, CO_2_ concentration, duration of exposure, species type, and nutritional outcomes.

### 2.1. PECO Components and Criteria

Population (P): We included studies explicitly focusing on kale (*Brassica oleracea* and all common names) and spinach (*Spinacia oleracea* and all common names), including various cultivars. Studies on other crops or combined datasets that did not provide disaggregated results for kale and spinach were excluded.

Exposure (E): We selected studies that investigated the impact of CO_2_ levels ranging from 650 ppm to over 3000 ppm. Studies were excluded if they combined CO_2_ exposure with other factors (e.g., drought or heat) without isolating CO_2_ effects.

Comparator (C): Only studies comparing CO_2_-exposed crops to controls grown under ambient CO_2_ conditions (approximately 400–450 ppm) were included. Studies lacking a proper control or baseline comparator were excluded.

Outcome (O): We focused on changes in nutritional constituents namely, carbohydrates, minerals, vitamins, nitrogenous compounds, yields, and photosynthetic parameters. Studies were required to report these outcomes for kale and spinach without aggregation with other crops.

### 2.2. Literature Search, Study Selection, and Data Extraction

The literature search was systematically conducted over a number of databases, including Scopus, ScienceDirect, Web of Science Core Collection, Google Scholar, CAB Abstracts, and PubMed, with searches concluding on 12 March 2024. The search strategy aimed to capture all relevant peer-reviewed articles examining eCO_2_ effects on kale and spinach across all years (i.e., all published studies till date were synthesised in this analysis). This search process resulted in the discovery of 872 studies. References were managed using Zotero (online version 6.0.35), which facilitated the removal of duplicates and the organisation of the literature. After removing 138 duplicates, 734 unique articles were filtered for PECO eligibility using the SysRev review tool (Insilica LLC, Rockville, MD, USA) where only titles and abstracts were assessed. Relevant articles (N = 42) were then subjected to a full-text review based on predefined PECO criteria (full details see Supplementary Table S1) as well as assessment of statistical rigour and completeness of data. Following several unsuccessful email requests, seven full texts could not be assessed. In addition, 29 studies were excluded after full-text review for not reporting the required outcomes (e.g., incomplete data, combined data on effects of eCO_2_ with other environmental stressors; lacking necessary statistical details; and relevant data could not be obtained from authors directly). Overall, 13 studies were retained, providing 346 effect sizes (full flow diagram: Supplementary Figure S1). The review was done in tandem by 2 reviewers (JUE and JOO), and any disputes were passed to a 3rd reviewer (SR). Data extraction was performed from text, tables, and figures of the selected studies, and MetaDigitize software (CRAN v1.0.1) was utilised to obtain data from graphs. At the same time, summary statistics were calculated from studies providing raw data via Excel. An outlier analysis was conducted, leading to the inclusion of only 339 effect sizes in the final meta-analysis (see Supplementary File S3 R Markdown File). Information on potential moderators such as species type, CO_2_ exposure duration, and outcome categories was also extracted and coded.

### 2.3. Effect Size and Variance Calculations

Hedges’ g [20] was used to calculate effect sizes., a measure that accounts for small sample sizes via the formula:

$$g=\frac{M_{1}-M_{2}}{{SD}_{pooled}}$$

where M_1_ and M_2_ represent the means of the CO_2_-exposed and control groups, respectively, while SD_pooled is the pooled standard deviation.

The pooled standard deviation is calculated using:

$${SD}_{pooled}=\sqrt{\frac{\left(n_{1}-1\right)\cdot s\frac{2}{1}+\left(n_{2}-1\right)\cdot s\frac{2}{2}}{n_{1}+n_{2}-2}}$$

with s_1_ and s_2_ representing the standard deviations and n_1_ and n_2_ the sample sizes. The Hedges’ correction factor (J) was applied as follows:

$$J=1-\frac{3}{4\left(n_{1}+n_{2}\right)-9}$$

The corrected effect size is:

$$g_{corrected}=g\cdot J$$

The variance of each effect size was calculated to estimate precision, with the standard error of g given by:

$${SE}_{\left(g\right)}=\sqrt{\frac{J^{2}(\frac{1}{n_{1}}+\frac{1}{n_{2}})+\frac{g^{2}}{2(n_{1}+n_{2})}}{n_{1}+n_{2}-2}}$$

More details of the calculations are found in Supplementary File S1, p. 6.

### 2.4. Statistical Analysis

Statistical analyses were carried out using R (version 4.3.3) with the {metafor} package (version 3.8.1) [21]. An overall meta-analytic model was constructed to estimate the general impact of eCO_2_ on nutritional quality across kale and spinach. This model employed a random-effects approach to account for variability among studies, assessed through the Q-test and I^2^ statistic [22].

Single-moderator analyses were performed to explore the influence of factors such as crop type, CO_2_ exposure levels, exposure duration, and species type on effect sizes. Multi-moderator models were developed to examine interactions between these factors and account for residual heterogeneity. Due to potential dependencies in effect sizes (e.g., multiple outcomes per study), multilevel mixed-effects models were used, incorporating random effects for study identifiers.

Outlier analysis and sensitivity checks ensured the robustness of findings (Figures S3 and S4). Publication bias was assessed using funnel plots (Supplementary Figures S5–S9 and S11–S15), and no substantial asymmetry was observed. Data visualisation was conducted using the orchaRd package (version 2.0.0) providing explicit graphical representations of effect sizes and confidence intervals [23]. Full methodological details, including data extraction and R scripts, are available in the Supplementary Files S1–S3.

## 3. Results

### 3.1. Bibliography Summary

**Figure 1:**
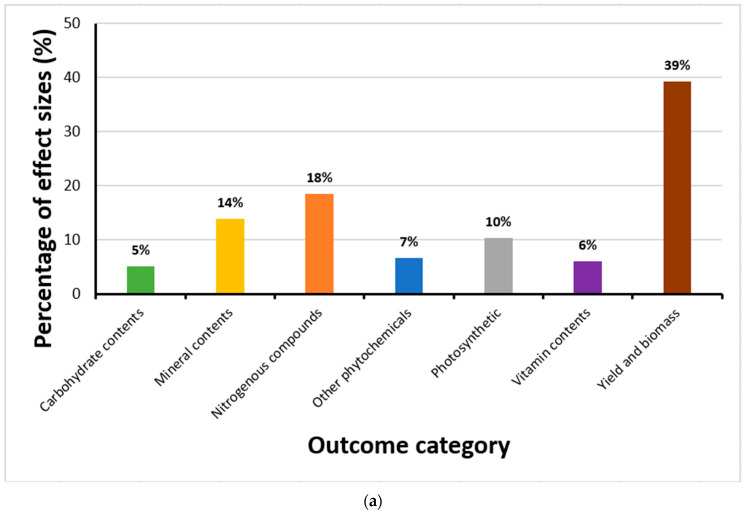

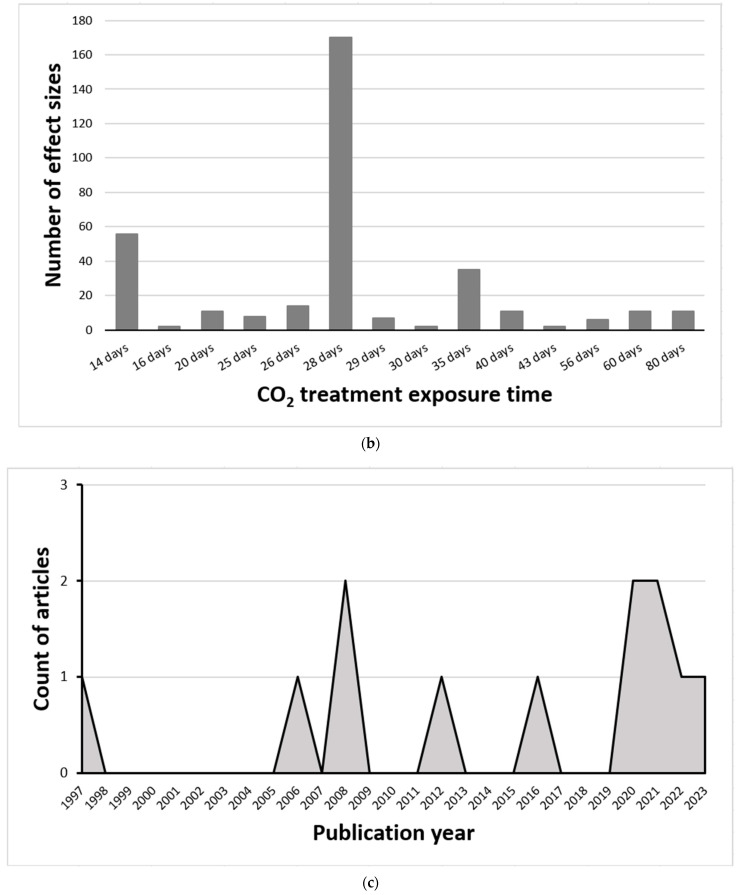
Distribution of effect sizes across outcome categories, exposure duration, and publication trends. (**a**) Proportion of effect sizes classified by outcome category, showing that yield and biomass changes accounted for 39%, nitrogenous compounds 18%, minerals 14%, photosynthetic parameters 10%, phytochemicals 7%, vitamins 6%, and carbohydrates 5%. (**b**) Frequency of effect sizes by CO_2_ exposure duration, with most studies conducted at 28 days (170 effect sizes), followed by shorter and longer durations ranging from 14 to 80 days. (**c**) Temporal trend in the number of published articles on eCO_2_ effects on kale and spinach from 1997 to 2024, indicating no significant increase in research output over time.

Overall, after the analyses of outliers, 339 effect sizes generated from 13 articles were retained. Most effect sizes at the time the studies were conducted (and effects evaluated) showed changes in yield and biomass (39%); a smaller percentage examined modifications in nitrogenous compounds (18%), with only a few being connected with mineral contents (14%), photosynthetic components (10%), phytochemicals (7%), vitamin contents (6%) or carbohydrate contents (5%) (Figure 1a).

**Figure 2:**
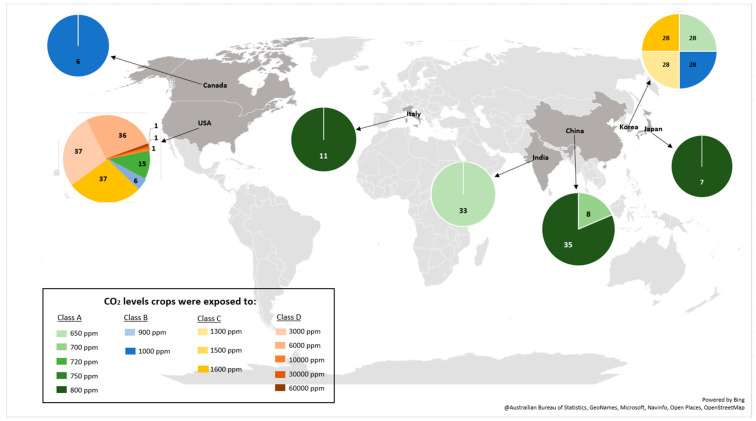
Geographical distribution of studies included in the meta-analysis and associated CO_2_ treatment levels. Map showing that most studies were conducted in the USA (CO_2_ levels: 720–60,000 ppm), followed by Korea (700–1600 ppm), China (700–800 ppm), India (650 ppm), Italy (800 ppm), Japan (800 ppm), and Canada (1000 ppm). The size of circles represents the number of effect sizes contributed by each country.

Of the effect sizes calculated, 170 were from crops exposed to eCO_2_ for 28 days with the remainder split between 14 days (56), 35 days (35), 26 days (14), 20 days (11), 40 days (11), 60 days (11), 80 days (11), 25 days (8), 29 days (7), 56 days (6), 16 days (2), 30 days (2) and 43 days (2) respectively (Figure 1b). The number of research articles published yearly did not appear to increase significantly from 1997 to 2024, as shown in Figure 1c. Furthermore, the geographic distribution of the research (Figure 2) shows that most were conducted in the United States of America. The CO_2_ levels crops were exposed to during the experiments in the USA were around 720–60,000 ppm; Korea 700–1600 ppm; China 700–800 ppm; India 650 ppm; Italy 800 ppm; Japan 800 ppm; and Canada 1000 ppm.

Seventy-nine per cent of the effect sizes originated from studies investigating the effect of eCO_2_ on kale, while the remaining twenty-one per cen, investigated spinach crops. The effect sizes associated with kale species were from *Brassica oleracea* cv. alboglabra Bailey (41%) followed by Palmifolia DC (20%), Virdis (20%), Sijicutiao (9%), acephala Winterbor F1 (5%), stem marrow kale (2%), Toscano (0.3%), Winterbor (0.3%) and unspecified (2%) cultivars, respectively. Most of the effect sizes were associated with spinach species were from unspecified cultivars (45%) with the remainder split between *Spinacea oleracea* cv. Gigante invernale (15%), Huangjia (15%), Wase Crone (10%), Melody (1%), Harmony (1%), Bloomsdale LS (1%) and *Ipomoea aquatica* cv. Forssk (11%) (see Supplementary Material Figure S2).

### 3.2. Overall and Combined Effect

**Figure 3:**
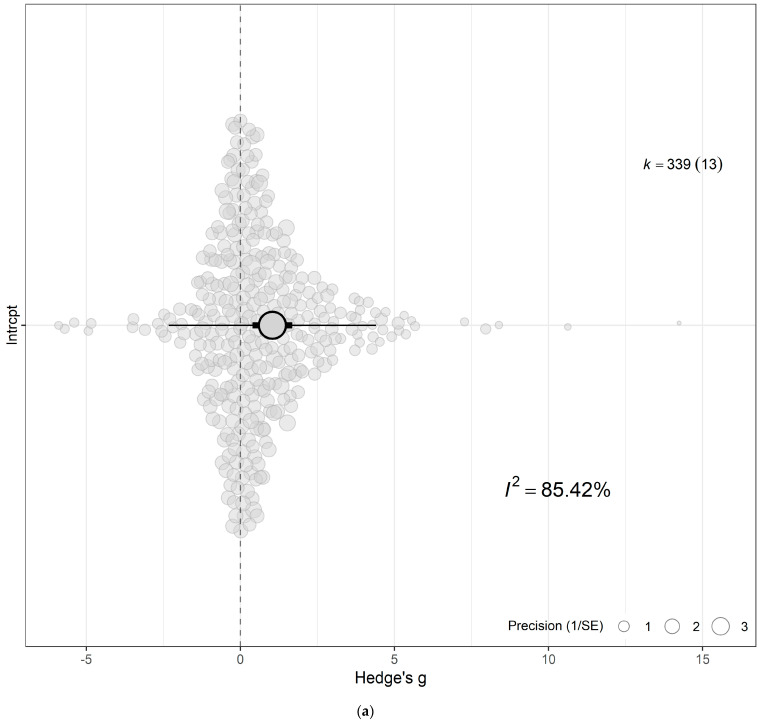

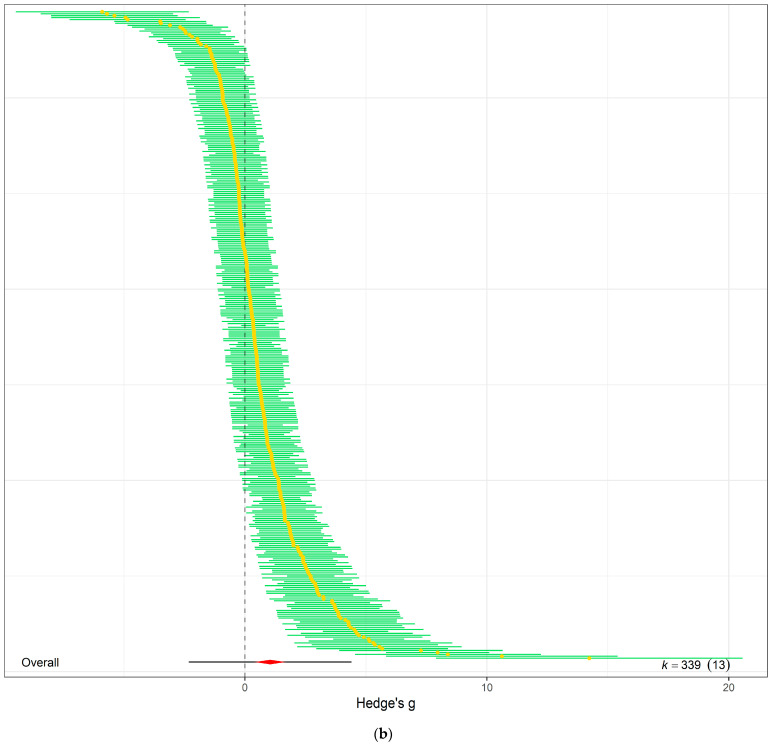
(**a**) The overall meta-analytic model demonstrates that eCO_2_ causes moderate combined increase in biomass and nutritional constituents of both kale and spinach crops (Hedges’ g value of 1.04, 95% CI [0.40, 1.69], t_12_ = 3.5125; *p* = 0.0043). In all orchard plots, individual effect sizes from studies are shown by the coloured bubbles in each figure, the estimated mean Hedges’ g values are represented by the circular dots, the 95% confidence intervals are represented by the strong error bars, and the 95% prediction interval is represented by the thin error bars. Each group’s impact size is represented by k, and the number of studies from which each effect size was derived is shown in brackets. (**b**) A caterpillar plot was also used to visualise the overall meta-analytic model. At the bottom, shown in red with a black 95% confidence interval bar, is the calculated mean Hedges’ g value. Each effect size is represented by a yellow dot with green 95% CI bars, and they are sorted by magnitude, drawing from 13 distinct publications and 339 effect sizes.

**Figure 4:**
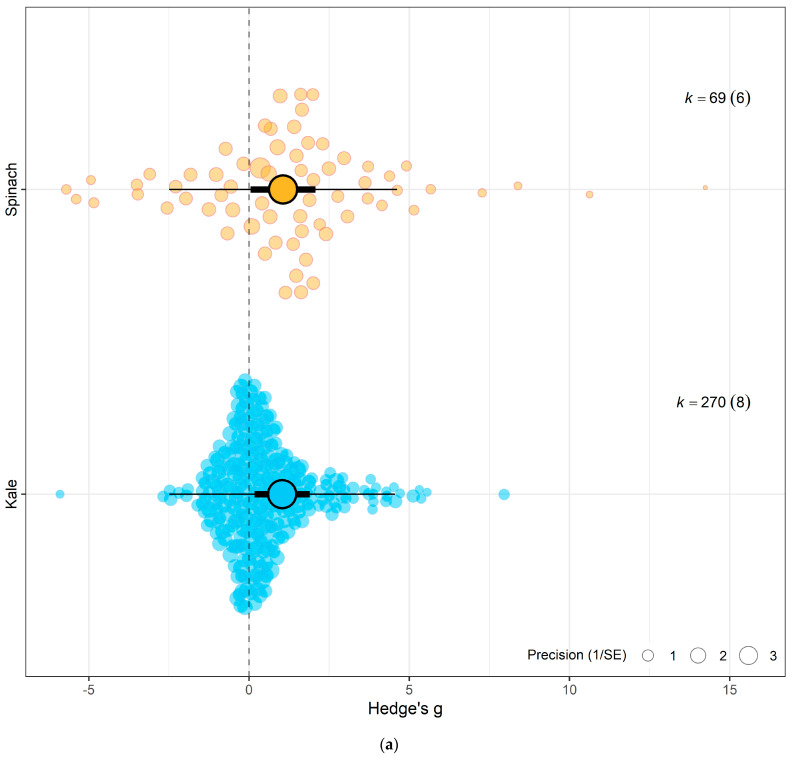

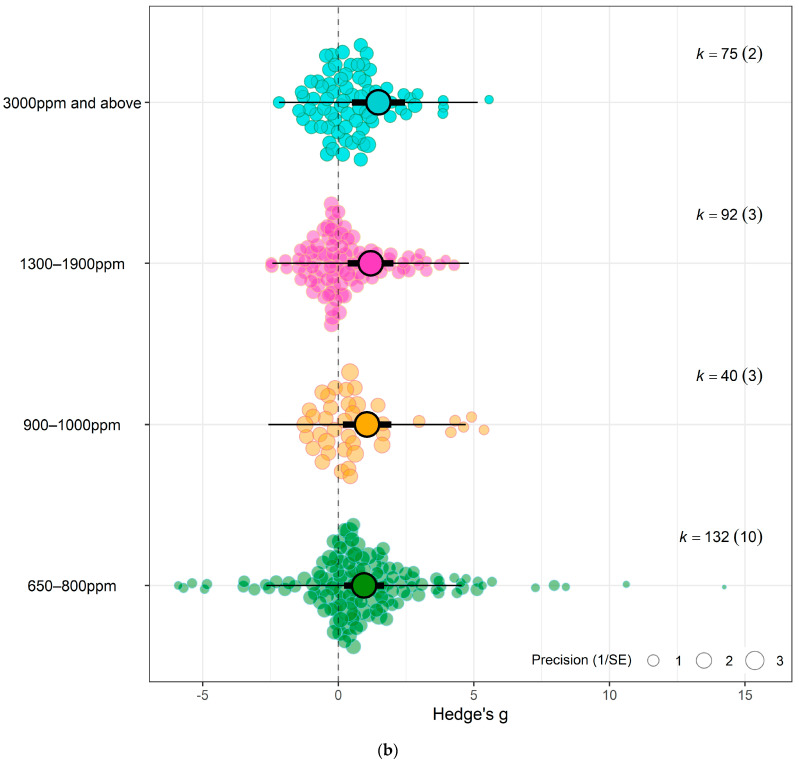

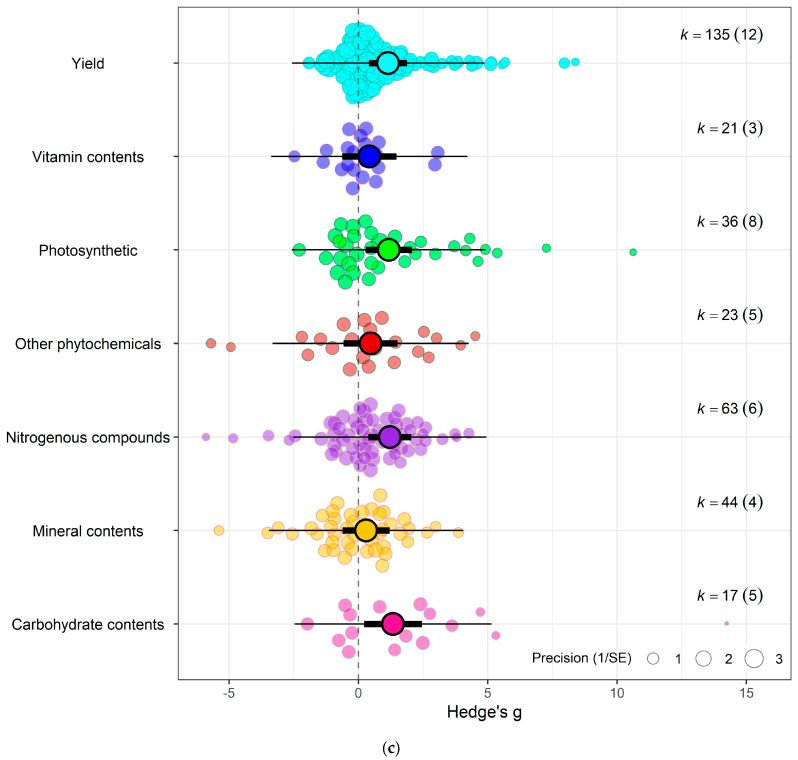

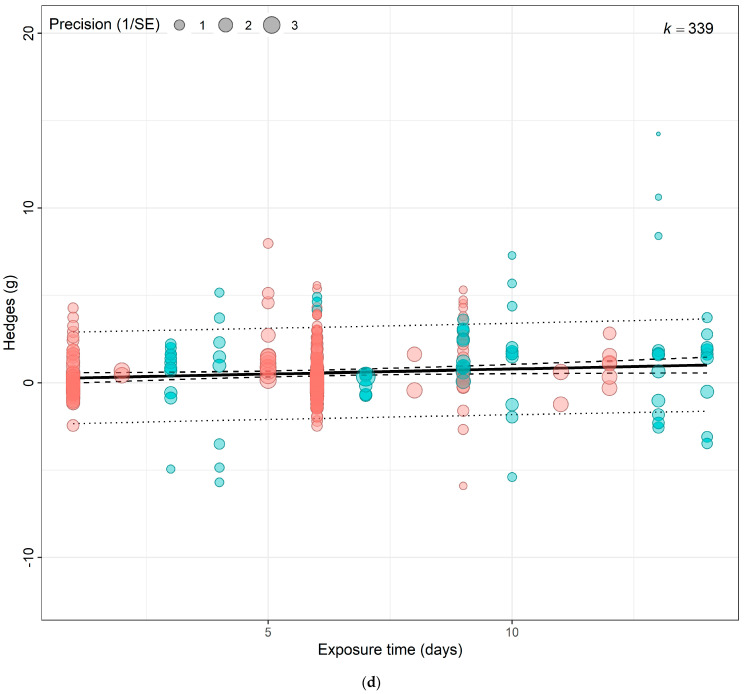
(**a**) Using crop type as moderator, the model shows that eCO_2_ causes more overall increase in biomass and nutritional constituents in exposed spinach crops (Hedges’ g = 1.06, CI = [0.05, 2.07]) compared to kale (Hedges’ g = 1.03, CI = [0.17, 1.90]) (t_337_ = 2.6321, *p* = 0.0233). (**b**) Using CO_2_ levels as moderator, the model show that the higher the CO_2_ level, the greater the combined increase in biomass and nutritional contents in both spinach and kale crops—650–800 ppm level (Hedges’ g = 0.95, CI = 0.21, 1.69, t_335_ = 2.9153, *p* = 0.0172), 900–1000 ppm (Hedges’ g = 1.06, CI = 0.16, 1.96, t_335_ = 3.2617, *p* = 0.7292), 1300–1900 ppm (Hedges’ g = 1.19, CI = 0.35, 2.04, t_335_ = 3.7860, *p* = 0.30846) and 3000 ppm > (Hedges’ g = 1.48, CI = 0.50, 2.46, t_335_ = 4.3399, *p* = 0.1552). (**c**) Using the outcome category as moderator, the model show that eCO2 causes increase in only yields (Hedges’ g = 1.15, CI = 0.41, 1.89, t_332_ = 2.4368, *p* = 0.6264), carbohydrates (Hedges’ g = 1.34, CI = 0.22, 2.46, t_332_ = 2.9541, *p* = 0.0265), nitrogenous compounds (Hedges’ g = 1.21, CI = 0.38, 2.04, t_332_ = 2.6259, *p* = 0.7657) and photosynthetic components (Hedges’ g = 1.18, CI = 0.28, 2.07, t_332_ = 2.5654, *p* = 0.7200). The changes in mineral (Hedges’ g = 0.30, CI = −0.61, 1.21, t_332_ = 0.4407, *p* = 0.0135), vitamins (Hedges’ g = 0.42, CI = −0.63, 1.48, t_332_ = 0.9707, *p* = 0.0516) and other phytochemicals (Hedges’ g = 0.47, CI = −0.57, 1.51, t_332_ = 1.0996, *p* = 0.0690) were not significant. (**d**) Bubble plot assessing whether the number of days plants were exposed to affect the yield and nutritional response to the effects of eCO_2_ (expressed as Hedges g), points coloured blue are spinach species while red are kale (t_1,13_ = 0.2363, *p* = 0.9397). The solid line represents the meta-regression estimate; dashed lines indicate the 95% confidence interval, and dotted lines indicate the 95% prediction interval.

The meta-analytic approach evaluating the combined effect of eCO_2_ unveiled a moderate increase in yield, biomass and nutritional components of kale and spinach crops exposed to atmospheric CO_2_ metrics ranging from 650 to 60,000 ppm compared with crops cultivated at ambient levels of 350–400 ppm (Hedges’ g = 1.04; CI 0.40, 1.69; *p* = 0.0043) (Figure 3a,b and Supplementary Table S2). Sizeable residual heterogeneity both between (I_between_^2^ > 33%) and within (I_within_^2^ > 54%) studies was apparent. The model was incrementally expanded to incorporate single moderators to identify important sources of response variability and evaluate the impacts of eCO_2_ across the types of crops, outcome category, specific constituents measured, period of exposure time and CO_2_ levels. Testing the combined interactive effect of eCO_2_ presence relative to crop types, group and CO_2_ levels revealed that a portion of the heterogeneity seen in the overall model was caused by the difference in crop type, level of CO_2_ and category of outcome comparator (QM_31,308_ = 3.7943, *p* < 0.001) (See Figure 4 and Supplementary Tables S3–S10).

Testing sub-group responses showed that eCO_2_ caused a slightly greater increase in overall yield and nutritional parameters of spinach crops (Hedges’ g = 1.06, CI = 0.05, 2.07) compared to kale crops (Hedges’ g = 1.03, CI = 0.17, 1.90) (Omnibus test of moderators: QM_2,337_ = 5.703, *p* = 0.0037) (Figure 4a). Across both crops types combined, only the following outcome category showed statistically significant difference—yields (Hedges’ g = 1.15, CI = 0.41, 1.89), carbohydrates (Hedges’ g = 1.34, CI = 0.22, 2.46), nitrogenous compounds (Hedges’ g = 1.21, CI = 0.38, 2.04) and photosynthetic parameters (Hedges’ g = 1.18, CI = 0.28, 2.07) with increases. While the changes observed in vitamins (Hedges’ g = 0.42, CI = −0.63, 1.48), mineral contents (Hedges’ g = 0.30, CI = −0.61, 1.21) and other phytochemicals (Hedges’ g = 0.47, CI = −0.57, 1.51) were not significant (Omnibus test of moderators detected significant differences (QM_7,337_ = 4.0457, *p* = 0.0003)) (Figure 4c). For the differences across CO_2_ levels, the model showed that as eCO_2_ level increased, there was a combined increased on yields and all nutritional parameters in both kale and spinach (Omnibus Test of Moderators (QM(df1 = 4, df2 = 335) = 3.3138, *p* = 0.0111) (Figure 4b). There was no significant changes or difference between the exposure time across all groups (Figure 4d).

**Figure 5:**
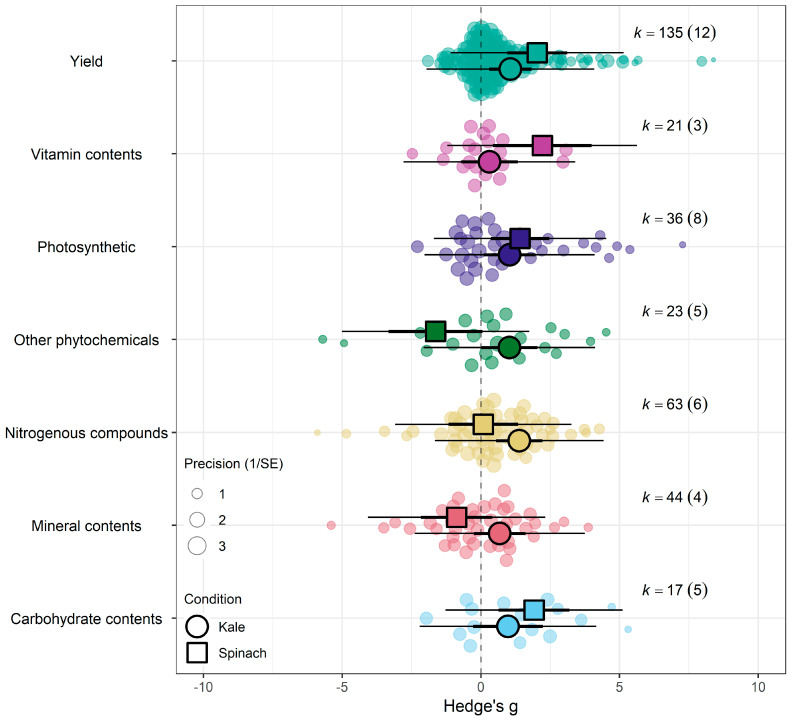
Orchard plot showing the combined effect of eCO_2_ on all crops with a complex model of crop type and outcome category as moderators.

When isolating the effect of eCO_2_ on kale crops only across the different outcome categories, there was significant increase in photosynthetic parameters, yields, nitrogenous compounds and other phytochemicals only. Similarly, spinach crops showed increase in photosynthetic parameters, yields, vitamin and carbohydrates contents. All other constituents showed no significant changes whether higher or lower (QM_6,263_ = 1.6922, *p* = 0.1231) (see Figure 5 and Supplementary Table S9).

### 3.3. Effect on Individual Constituents

**Figure 6:**
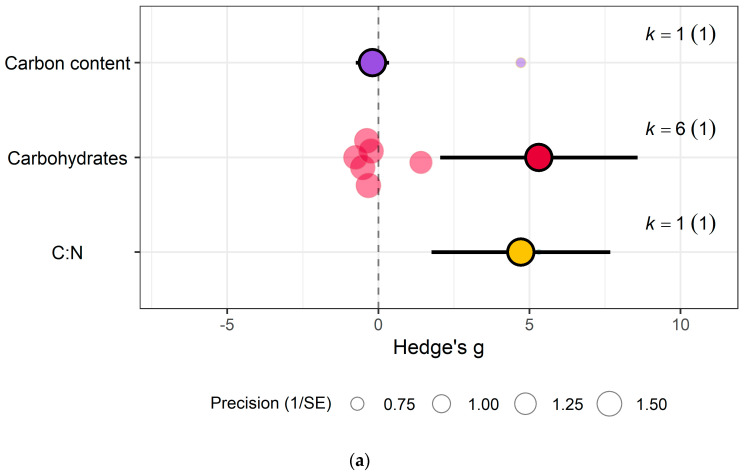

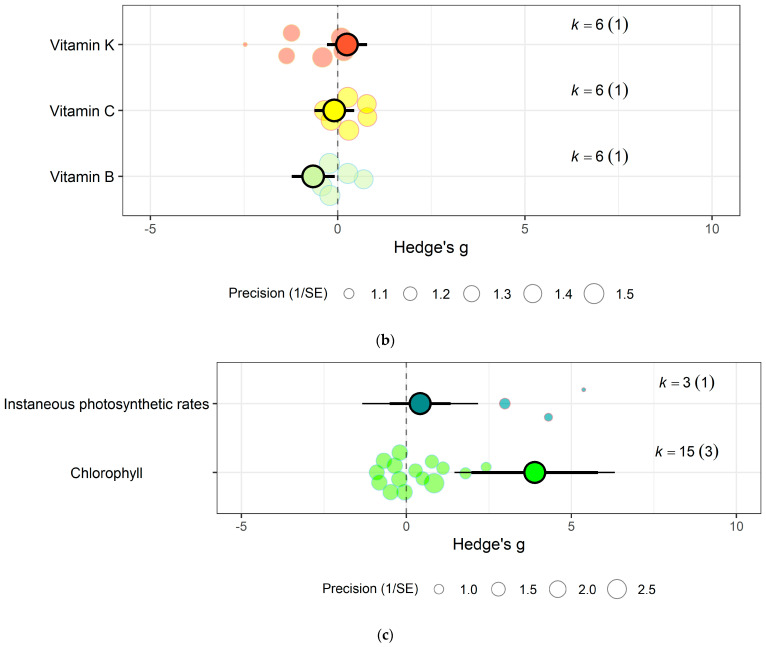

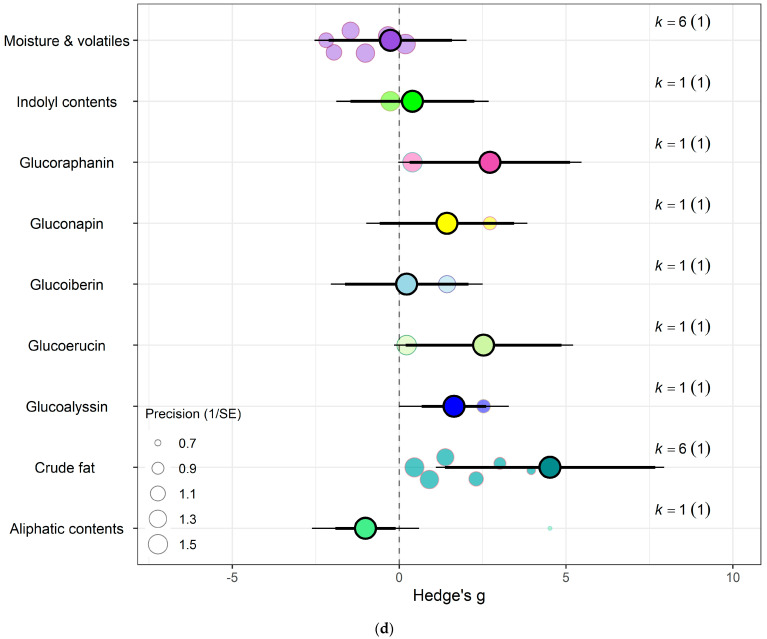

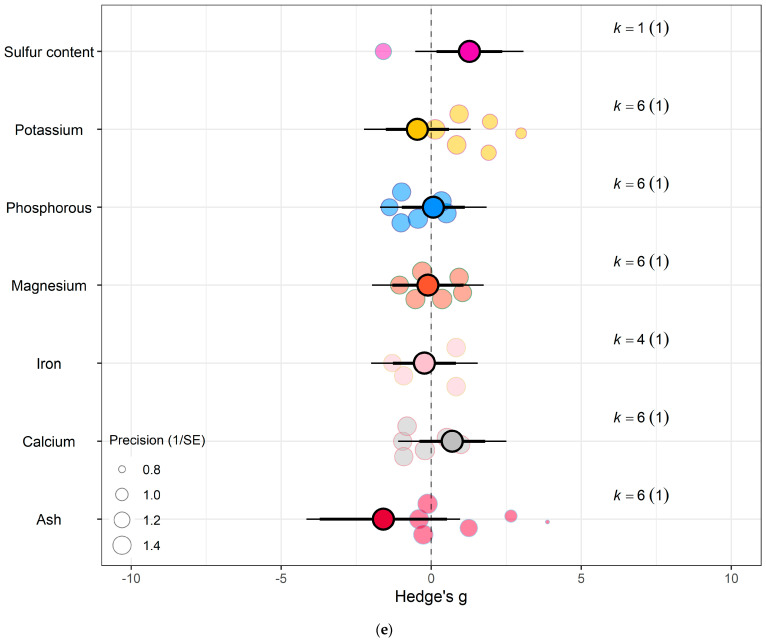

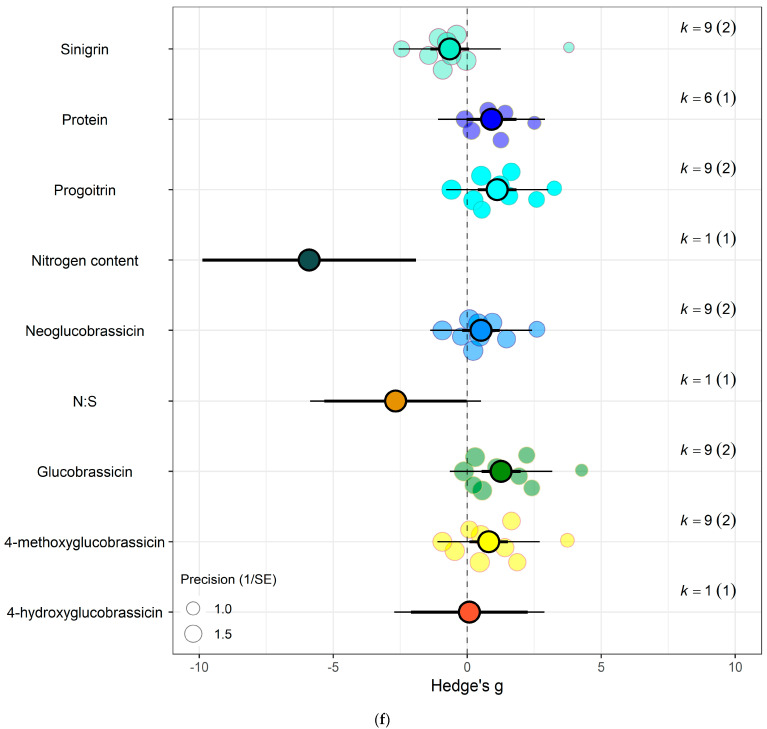

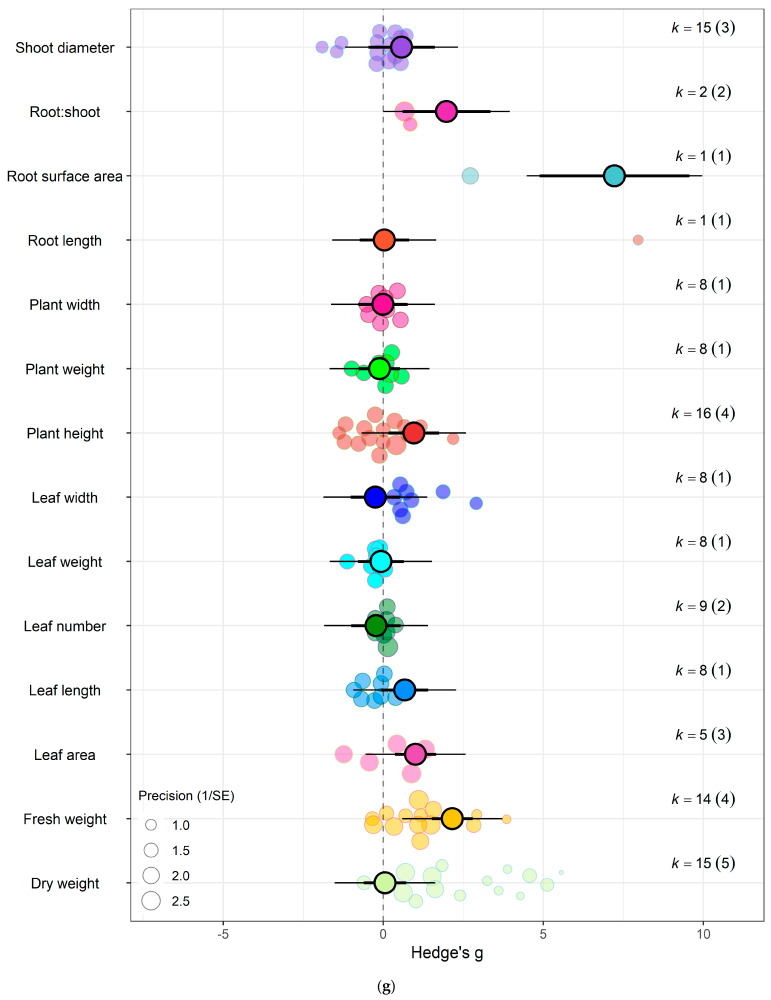
For kale crops only (**a**) Effect of eCO_2_ on carbohydrate contents with constituent type as a moderator. (**b**) Effect of eCO_2_ on vitamin contents only. (**c**) Effect of eCO_2_ on photosynthetic components contents. (**d**) Effect of eCO_2_ on non-classed phytochemical contents. (**e**) Effect of eCO_2_ on mineral contents. (**f**) Effect of eCO_2_ on nitrogenous compounds. (**g**) Effect of eCO_2_ on yield and biomass components.

**Figure 7:**
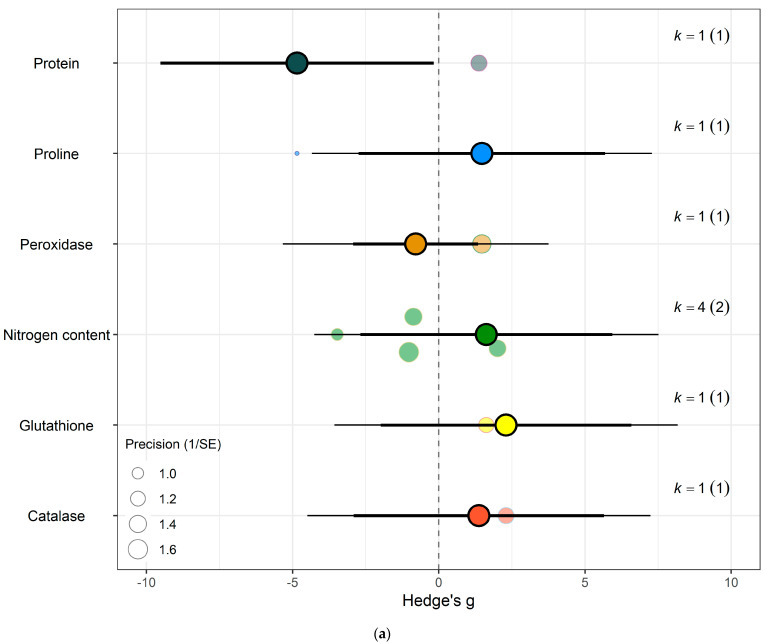

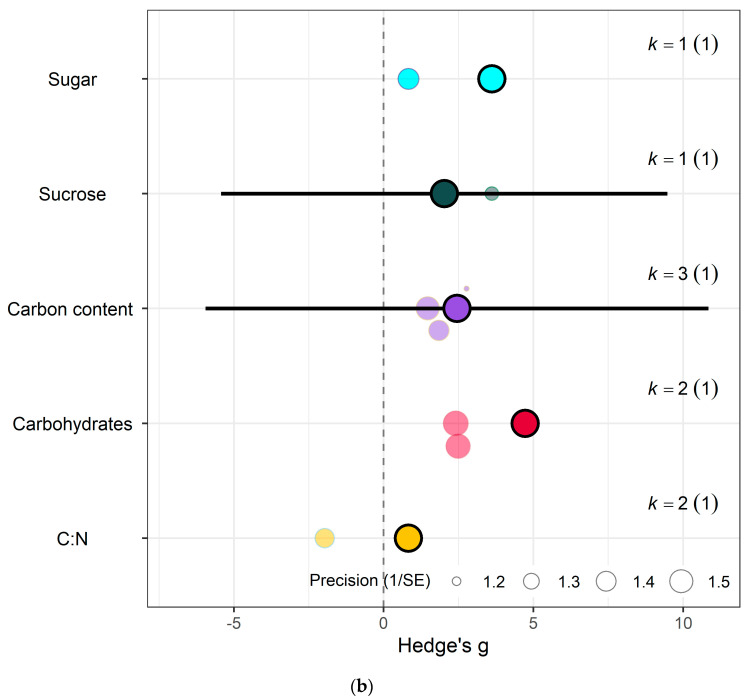

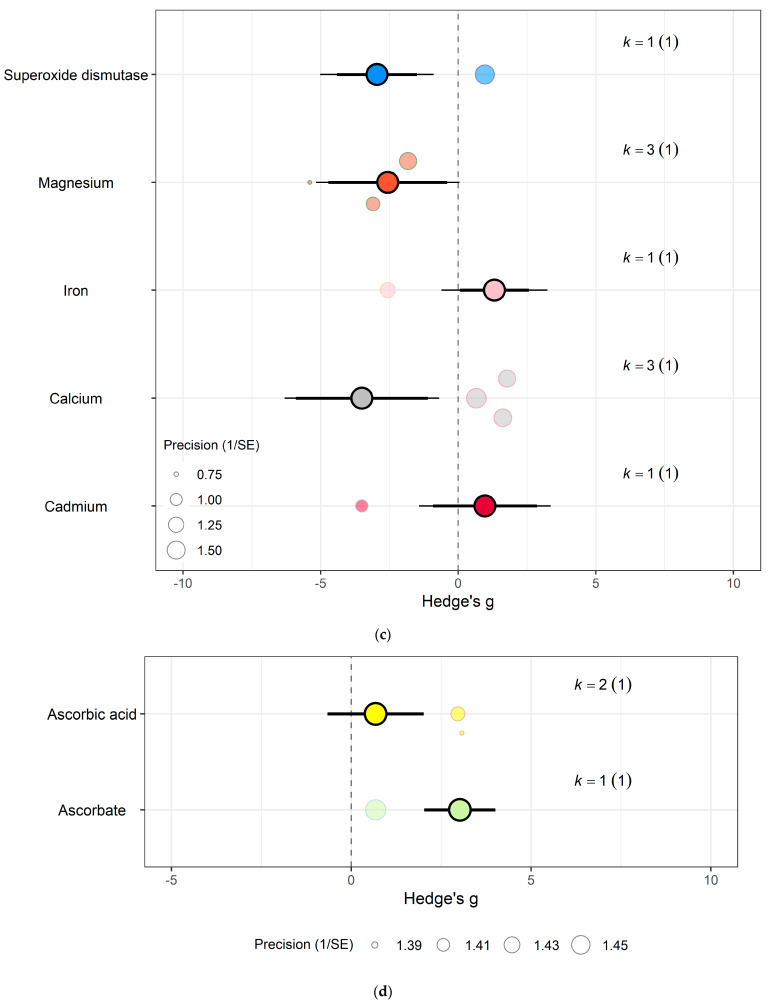

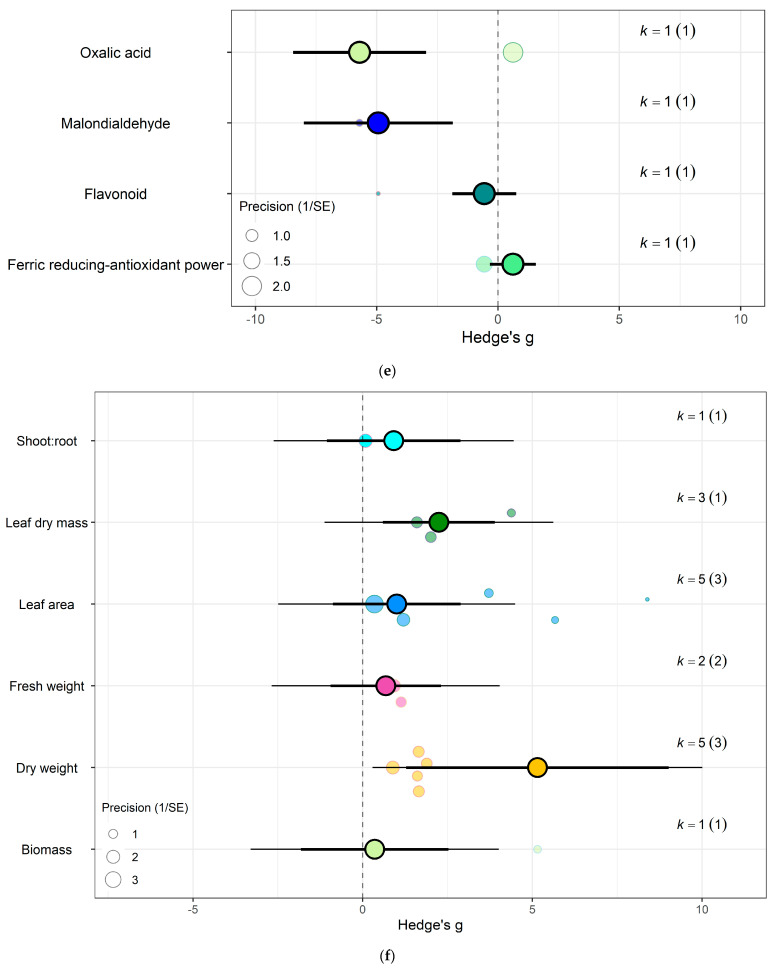

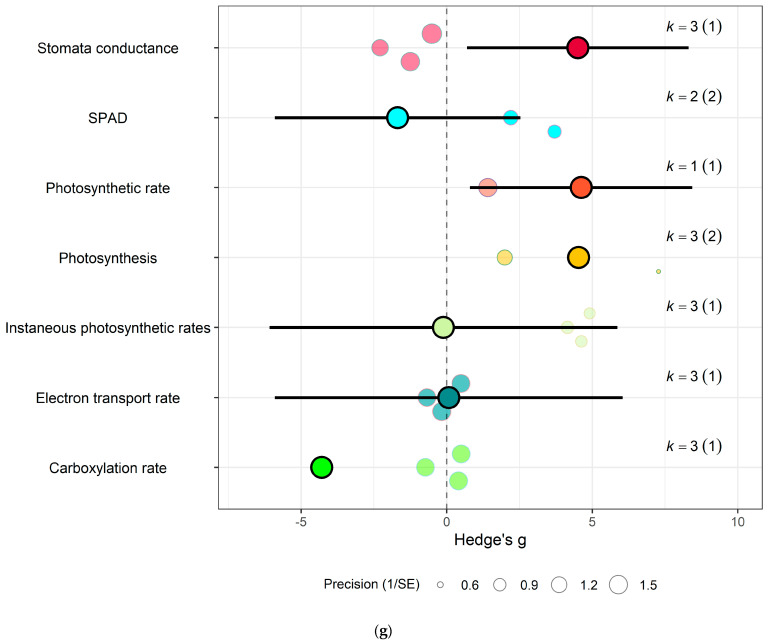
For spinach crops only (**a**) Effect of eCO_2_ on nitrogenous compounds with constituent type as a moderator. (**b**) Effect of eCO_2_ on carbohydrate contents. (**c**) Effect of eCO_2_ on mineral contents. (**d**) Effect of eCO_2_ on vitamin contents. (**e**) Effect of eCO_2_ on non-classed phytochemical contents. (**f**) Effect of eCO_2_ on yield components. (**g**) Effect of eCO_2_ on photosynthetic components.

The impact of eCO_2_ varied according to the type of specific constituents measured. Within yields category of kale crops, only fresh weight (Hedges’ g = 2.15, CI = 1.52, 2.79), leaf area (Hedges’ g = 1.00, CI = 0.36, 1.65), plant height (Hedges’ g = 0.95, CI = 0.17, 1.77), root surface area (Hedges’ g = 7.22, CI = 4.89, 9.56) and root-shoot ratio (Hedges’ g = 1.98, CI = 0.60, 3.35) showed increases to eCO_2_ (Figure 6g) While eCO_2_ increased only dry weight (Hedges’ g = 5.15, CI = 1.28, 9.02) and leaf dry mass (Hedges’ g = 2.24, CI = 0.60, 3.90) in spinach crops (Figure 7f) (QM_14_ = 121.5006, *p* < 0.0001).

In the carbohydrate category, eCO_2_ caused an increase within kale crops only in the carbon-nitrogen ratio (Hedges’ g = 4.72, CI = 1.76, 7.68) and unclassified carbohydrate contents (Hedges’ g = 5.31, CI = 2.04, 8.58) (Figure 6a,b) (QM_3_ = 20.3902, *p* = 0.0001). All other specific measured constituents did not show any significant changes (increase or decrease).

For protein and nitrogenous compounds, kale showed increases in 4-methoxyglucobrassin (Hedges’ g = 0.80, CI = 0.08, 1.52), glucobrassin (Hedges’ g = 1.26, CI = 0.54, 1.99) and progoitrin (Hedges’ g = 1.12, CI = 0.40, 1.84) but decrease in nitrogen-sulphur ratio (Hedges’ g = −2.68, CI = −5.33, −0.02) and unclassified nitrogen contents (Hedges’ g = −5.90, CI = −9.89, −1.91) (Figure 6f). While spinach showed decrease in unclassified proteins (Hedges’ g = −4.85, CI = −9.52, −0.17) (Figure 7a) (QM_9_ = 46.8107, *p* < 0.0001).

Sulphur contents under the mineral category was significantly decreased in kale crops (Hedges’ g = 1.27, CI = 0.18, 2.37) (Figure 6e). While for spinach crops showed decrease in calcium (Hedges’ g = −3.50, CI = −5.90, −1.10), magnesium (Hedges’ g = −2.56, CI = −4.71, −0.40), superoxide dismutase (Hedges’ g = −2.95, Ci = −4.40, −1.50) but increase in iron (Hedges’ g = 1.32, CI = 0.06, 2.57) (Figure 7c) (QM_7_ = 15.4177, *p* = 0.0310). There were no significant changes for all other specific measure constituent.

Similarly, eCO_2_ caused an increase in chlorophyll values (Hedges’ g = 3.89, CI = 1.97, 5.80) of kale crops only (Figure 6c). While spinach showed increase in photosynthetic rates (Hedges’ g = 4.62, CI = 0.80, 8.44) and stomata conductance (Hedges’ g = 4.51, CI = 0.70, 8.31) but decrease in carboxylation rate (Hedges’ g = −4.29, CI = −8.37, −0.21) (Figure 7g) (QM_2_ = 16.5623, *p* = 0.0003). Kale crops showed decrease in vitamin B (Hedges’ g = −0.65, CI = −1.23, −0.08) (Figure 6b) while spinach showed increase in ascorbate (Hedges’ g = 3.02, CI = 2.03, 4.01) (Figure 7d) (QM_1_ = 4.9116, *p* = 0.0267).

For other phytochemical constituents, spinach crops showed only decreases and they were in malondialdehyde (Hedges’ g = −4.94, CI = −8.01, −1.86) and oxalic acid (Hedges’ g = −5.70, CI = −8.44, −2.96) (Figure 7e). While kale showed decrease in aliphatic contents (Hedges’ g = −1.01, CI = −1.91, −0.10) but increase in crude fat (Hedges’ g = 4.52, CI = 1.37, 7.68), glucoalyssin (Hedges’ g = 1.64, CI = 0.67, 2.61), glucoerucin (Hedges’ g = 2.53, CI = 0.19, 4.87) and glucoraphanin (Hedges’ g = 2.72, CI = 0.32, 5.12) (Figure 6d) (QM_9_ = 38.0996, *p* < 0.0001). Other specific measured constituents did not show any significant changes (also see Supplementary Table S9).

## 4. Discussion

### 4.1. Summary of the Combined Effect of Elevated CO_2_ on Spinach and Kale

The overall findings of this meta-analysis revealed a significant combined moderate increase in yield, biomass and nutritional contents in spinach and kale grown under eCO_2_ conditions compared to ambient equivalents, with a Hedges’ g value of 1.04 (*p* = 0.0043). This aligns with prior studies demonstrating the stimulating effect of eCO_2_ [24]. However, the meta-analysis also identified significant variability, suggesting that the response to eCO_2_ is not uniform and varies across different crops, cultivars, CO_2_ levels and constituents measured [18]. Spinach exhibited a stronger response to eCO_2_ compared to kale, consistent with studies showing species-specific differences in photosynthetic efficiency and nitrogen use efficiency [25,26].

Although cultivar effects could not be fully resolved, the variability observed in *Brassica oleracea* cultivars suggests that genetic traits influence responsiveness to eCO_2,_ as also shown in other crops [27,28,29,30]. No significant differences were detected across exposure durations, possibly due to the short timeframe of most studies, which may not capture longer-term acclimation processes [15,17]. The positive correlation between higher CO_2_ concentrations and increased effect size highlights the need to consider concentration-specific responses when modelling future crop performance [31,32].

### 4.2. Effect on Nutritional Components and Implications for Global Health

(i) Yields and Biomass

Elevated CO_2_ was found to significantly increase yields and biomass in spinach and kale, particularly in parameters such as root surface area, plant height, fresh weight, and leaf dry mass. This aligns with numerous studies that have documented similar increases in biomass and yield under eCO_2_ across various crop species [33]. The enhancement in biomass is primarily driven by increased photosynthetic rates and reduced photorespiration, which are direct responses to higher atmospheric CO_2_ [32]. However, the magnitude of yield increases can be variable depending on other factors such as nutrient availability, water supply, and cultivar characteristics [34]. In particular, the increase in biomass does not always translate into proportional increases in yield quality, as changes in nutrient content can occur simultaneously [35].

The impact of eCO_2_ on crop quality also has broader implications for global food security. As crop yields increase under eCO_2_, the dilution effect, where higher biomass dilutes the concentration of nutrients, could lead to a paradox where more food is produced but with lower nutritional value. This could exacerbate micro-deficiency problem, where people have enough calories to eat but lack essential nutrients, leading to malnutrition [36]. In addition, the variability in response among different crops suggests that some regions may be more adversely affected than others, depending on their primary food sources. This could lead to greater disparities in nutritional outcomes, with populations in certain areas experiencing more severe nutrient deficiencies.

(ii) Photosynthetic Parameters

The meta-analysis indicated that eCO_2_ significantly enhanced several photosynthetic characteristics, including stomatal conductance, photosynthetic rate, and chlorophyll concentration in kale and spinach. These findings are consistent with the well-documented effect of eCO_2_ in increasing the photosynthetic capacity of plants by enhancing the carboxylation efficiency of RuBisCO (the enzyme ribulose-1,5-bisphosphate carboxylase/oxygenase) and reducing photorespiration [37]. The increase in photosynthetic activity under eCO_2_ often leads to higher biomass production, as observed in this study. However, the relationship between photosynthetic enhancement and yield is not always straightforward, as factors such as nutrient availability, water use efficiency, and the balance between source and sink capacities can modulate the overall growth response [17]. In spinach, the enhanced photosynthesis was reflected in increased biomass, but in kale, the response was more variable, suggesting that other physiological or environmental factors may be limiting the full realisation of the CO_2_ fertilisation effect.

(iii) Carbohydrate/carbon contents

The analysis showed that eCO_2_ led to a significant increase in the carbon-nitrogen ratio and unclassified carbohydrate contents in kale only, whereas no significant changes were observed in other measured constituents in both kale and spinach. The demonstrated increase is consistent with the general understanding that eCO_2_ stimulates carbohydrate synthesis through enhanced photosynthetic carbon fixation [38]. And increased carbohydrate accumulation under eCO_2_ has been reported in various crops, including wheat, rice, and maise, often leading to higher starch and sugar content [30]. However, this increase in carbohydrates can be accompanied by a reduction in protein concentration, leading to a dilution effect that could impact the nutritional quality of crops [39]. This trade-off between carbohydrate enrichment and protein reduction is a critical consideration for the nutritional implications of future crops grown under elevated CO_2_ conditions. Moreso, while increased carbohydrate accumulation has been well-documented in staple crops such as wheat, rice, and maize [30], our results suggest that leafy vegetables may exhibit a more selective response, with only certain carbohydrate fractions being affected.

Higher sugar contents, which may first seem to be advantageous for producing biomass in plants, have concerning implications for nutrition. Studies have indicated that increased crop sugar levels, especially in frequently consumed food crops, may be a factor in the rising rates of obesity and associated metabolic diseases like diabetes in developed nations [40,41]. In the United States, for example, the growth in carbohydrate content under eCO_2_ conditions may cause people to consume identical amounts of food but with considerably more sugar [42]. According to the CDC, more than 42% of adult Americans are obese, and more than 11% of people have diabetes [40]. The obesity pandemic could further be exacerbated by this “hidden-sugar” effect, which can lead to an increase in calorie intake without a commensurate gain in satiety. However, evidence in this area remains limited, and significant gaps in the literature highlight the need for further research.

(iv) Proteins and Nitrogenous Compounds

The meta-analysis found that nitrogenous compounds, particularly nitrogen-sulphur ration and other unclassified nitrogen contents, showed a significant decrease under eCO_2_, which is indicative of a potential decline in protein content. This is in line with numerous studies showing that eCO_2_ often leads to reduced nitrogen concentration in plant tissues, likely due to a combination of factors such as dilution by increased carbohydrate content and reduced nitrogen uptake efficiency [43]. The reduction in protein content under eCO_2_ has been a consistent finding across various crop species, including cereals and legumes, which raises concerns about the potential impact on human nutrition, especially in regions where plant-based diets are predominant [44,45]. The mechanisms underlying this reduction are complex and may involve changes in root-to-shoot nitrogen allocation, altered nitrogen assimilation pathways, and interactions with other environmental stressors such as drought or nutrient limitation [46].

The decline in protein content is particularly concerning given that billions of people worldwide rely on plant-based sources for their protein intake, especially in developing countries where animal protein is less accessible [45,47]. A reduction in protein content could exacerbate issues of malnutrition, leading to increased rates of stunting, impaired cognitive development, and weakened immune systems among vulnerable populations [2]. Moreover, the decline in protein quality, indicated by a reduction in essential amino acids, could further impact dietary adequacy. Studies have shown that eCO_2_ can lead to reductions in lysine and other essential amino acids in staple crops like wheat, rice, and soybeans, which are crucial for maintaining muscle mass, enzyme function, and overall metabolic health [31].

(v) Minerals

Mineral contents, including calcium, magnesium, and sulphur, showed varied responses to eCO_2_ in spinach and kale. While sulphur contents in kale showed a moderate increase, other minerals, such as calcium and magnesium, exhibited significant decrease in spinach. This differential response is consistent with previous research indicating that eCO_2_ can alter the nutrient composition of crops, often leading to reductions in essential minerals [48]. The decline in mineral content under eCO_2_ is thought to be related to the dilution effect, where the increased biomass production leads to lower concentrations of minerals per unit of plant tissue [35]. Additionally, changes in soil chemistry and nutrient uptake dynamics under eCO_2_ could contribute to these effects [35].

The observed decline in essential minerals such as calcium and magnesium in kale and spinach grown under eCO_2_ has severe implications for global nutrition as they exacerbate micronutrient deficiencies [36]. Reduction in minerals in crop produce contributes to the global problem of “hidden hunger” (a characteristic of micronutrient deficits), which the World Health Organization has identified as a significant worldwide health concern, especially in low- and middle-income nations [49]. Over two billion people worldwide suffer from hidden hunger, especially in low-income areas [6]. According to the most recent global assessment conducted by WHO, over 30% of people worldwide lack sufficient iron, and roughly 17% need zinc [49]. Micronutrients are vital for bone health, immune function, and metabolic processes, and deficiency has serious negative effects on health, including compromised immune systems, decreased cognitive development, and increased susceptibility to infections. The reduction in calcium and iron content due to eCO_2_ has been associated with a rise in the occurrences of anaemia, osteoporosis, and other related health problems [5]. Given that crop nutritional quality is declining under eCO_2_, these numbers are projected to increase.

(vi) Vitamins

Vitamin responses to eCO_2_ were variable, with vitamin B showing a significant decrease and ascorbate increase in this meta-analysis. The impact of eCO_2_ on vitamin content has received less research than other nutrients, but existing research suggests that the effects can be highly specific to the type of vitamin and crop species [50]. For instance, studies on rice and wheat have reported both increases and decreases in various vitamins under eCO_2_, with factors such as cultivar, soil conditions, and interactions with other environmental variables playing critical roles [24]. The findings in spinach and kale suggest that while some vitamins may benefit from eCO_2_, others could decline, potentially affecting the overall nutritional quality of these crops. Additionally, any decline in vitamin contents could have implications on global nutrition because kale and spinach are recognised for their high concentrations of vitamins A, C, and K, making them key contributors to daily nutritional requirements, particularly for maintaining vision, skin health, and blood coagulation [6].

(vii) Other Phytochemicals

Phytochemicals such as glucosinolates showed a significant decrease under eCO_2_, with compounds like glucoalyssin, glucoerucin, and glucoraphanin exhibiting a decline in kale. In contrast, kale also showed an increase in aliphatic glucosinolate content, while spinach exhibited a significant decrease in malondialdehyde and oxalic acid. This is in line with findings from prior research that have revealed changes in specific phytochemicals under eCO_2_, possibly as a response to altered carbon allocation and secondary metabolite synthesis pathways [47].

The response of phytochemicals to eCO_2_ is intricate and subject to change based on the specific compound and crop species. For instance, some studies have reported increased concentrations of flavonoids and phenolic compounds under eCO_2_, while others have found reductions [2,8,46]. These changes in phytochemical content can have significant implications for the health benefits of crops, as many phytochemicals are known for their antioxidant, anti-inflammatory, and anti-carcinogenic properties [8]. The increase in aliphatic glucosinolates in kale, for example, could enhance its cancer-preventive properties, while the decline in glucoalyssin, glucoerucin, and glucoraphanin may reduce its overall health-promoting potential.

Phytochemicals play a crucial role in the prevention of long-term illnesses such as cancer, diabetes, and cardiovascular disease. The findings of this meta-analysis indicate that eCO_2_ can alter the concentration of these compounds in crops, with some increasing while others decrease. This variability could affect the protective health benefits that these crops offer [6]. For instance, while the increase in aliphatic glucosinolates in kale may enhance its anti-cancer properties, the decline in key glucosinolates, crude fat, malondialdehyde, and oxalic acid suggests that eCO_2_ induces complex biochemical trade-offs that may influence the nutritional and functional properties of both kale and spinach [8].

The long-term implications of eCO_2_ on global health are profound, particularly as they relate to human nutrition and food security. As the meta-analysis has shown, while eCO_2_ can lead to increased crop yields, the quality of these crops specifically, their nutritional content, can be adversely affected. This poses significant challenges for global health, especially in regions where plant-based diets are predominant, and nutrient deficiencies are already a concern.

### 4.3. Implications for Future Sustainability and Agriculture

Long-term FACE experiments in the USA, such as those conducted on soybeans, wheat, and rice, have provided critical insights into the sustained impact of eCO_2_ on crops. These studies have demonstrated that while initial increases in photosynthesis and growth are common, these benefits can diminish over time due to nutrient limitations, particularly nitrogen [26]. For example, the SoyFACE project in Illinois has shown that while soybean yields increase under eCO_2_, the protein content decreases, posing challenges for both human nutrition and livestock feed [5]. The long-term nature of these studies also reveals the potential for acclimation effects, where plants may initially respond positively to eCO_2_, but over time, the benefits plateau or even decline. This acclimation is often linked to limitations in other resources, such as water and nutrients, highlighting the need for integrated approaches that consider the full range of environmental factors affecting crop growth [51].

Given the findings of both this meta-analysis and long-term FACE studies, there is a clear need for sustainable agricultural practices that can mitigate the negative impacts of eCO_2_ on crop nutrition. Soil nutrient management will be crucial in this regard. Practices such as precision agriculture, which optimises nutrient application based on real-time monitoring of crop needs, could help maintain or even improve the nutritional quality of crops under eCO_2_ conditions [18]. In addition, biofortification particularly, breeding crops to increase their nutrient content, could play a key role in addressing the reductions in essential minerals and vitamins observed under eCO_2_. This approach has already shown success in increasing the iron and zinc content of staple crops like rice and wheat and could be extended to other crops affected by eCO_2_ [47].

Diversified cropping systems, which involve growing a variety of crops rather than relying on monocultures, could also enhance resilience to the impacts of eCO_2_. By incorporating crops that are less sensitive to eCO_2_-induced nutritional changes, farmers can reduce the risk of nutrient deficiencies in their produce [52]. Moreover, integrating traditional and indigenous crop varieties, which may have unique adaptations to local environmental conditions, could provide additional resilience against the challenges posed by climate change and eCO_2_ [26]. The challenges posed by eCO_2_ for agriculture are multifaceted, involving not only the direct effects on crop yields and nutritional quality but also broader implications for agricultural sustainability, food systems, and global food security.

### 4.4. Limitations, Research Gaps, and Future Directions

This meta-analysis presents several limitations that must be considered in interpreting its findings. First, the scope of available data for spinach and kale under eCO_2_ conditions remains limited, with only 13 primary studies available, most of which were conducted in controlled environments within temperate regions. This restricts the ability to generalise the findings globally as crop responses could strongly be influenced by local factors such as soil properties, water availability, climate and management practices. Future research from regions outside the temperate zone and varying environmental stressors such as drought, heat, and nutrient availability, is needed to improve global relevance.

Second, substantial heterogeneity was observed across studies largely due to variability in experimental designs including differences in CO_2_ concentrations, exposure duration, nutrient and nutrient availability, water supply, growth conditions, and methods to quantify biomass and nutritional traits [38]. Although moderator analyses were applied to account for some of this variation, inconsistent reporting and lack of standardised protocols limited further resolution of these effects [51]. This highlights the need for more harmonised experimental approaches and improved data reporting in future eCO_2_ studies.

Third, a key challenge is the relatively short growing periods for these annual and biennial crops, meaning that studies are inherently limited to short-term effects of eCO_2_. This limits our understanding of how repeated seasonal exposures might alter crop physiology or nutritional quality over time. However, given the life cycle of kale and spinach, conducting truly long-term studies remains a practical challenge.

There is also a possibility of publication bias, as studies reporting significant effects of eCO_2_ on yield and nutrient composition may be more likely to be published than those reporting null responses. Although this meta-analysis employed funnel plots to assess for such bias, it cannot entirely eliminate the possibility that underrepresented studies may impact the synthesis of the results.

Importantly, interactions between eCO_2_ and other environmental stressors such as heat, drought, and nutrient limitation, remain underexplored for leafy vegetables These combined stresses are likely to define future growing conditions and may amplify or offset the effects observed under elevated CO_2_ alone [43]. Furthermore, the broader nutritional and health implications of eCO_2_-induced changes in crop quality, particularly reductions in essential nutrients and phytochemicals are still poorly quantified. Linking agronomic and nutritional data with epidemiological evidence would help clarify the potential consequences for human health, especially in populations heavily reliant on plant-based diets [46].

Future research should therefore prioritise multi-factor experiments, broader geographic coverage, and integration of crop physiology with nutritional and public health perspectives. Such approaches will be essential for developing effective mitigation strategies that balance productivity gains with the preservation of nutritional quality under rising atmospheric CO_2_ [48,52,53].

## 5. Conclusions

This meta-analysis highlights both opportunities and challenges posed by eCO_2_ for agricultural productivity and global nutrition. It demonstrates that eCO_2_ can enhance the biomass and yield of both spinach and kale, with spinach showing stronger positive response than kale. However, these gains in productivity are frequently accompanied by changes in nutritional composition, particularly reductions in protein and selected mineral contents indicating a trade-off between yield and nutritional quality under eCO_2_ conditions. The findings confirm that responses to elevated CO_2_ are not uniform and vary according to crop species, constituent type, and CO_2_ concentration. While increases in photosynthetic activity and carbohydrate-related traits under eCO_2_ may support greater biomass accumulation, these changes do not consistently translate into improved nutritional value. The observed declines in nitrogenous compounds and essential minerals highlight the risk of nutrient dilution in leafy vegetables that contribute substantially to dietary micronutrient intake. These results reinforce the need to evaluate future food security not only in terms of yield but also with respect to nutritional adequacy. Looking ahead, further research should prioritise multi-factor studies that examine the combined effects of elevated CO_2_ with other climate-related stressors, including heat, water limitation, and soil nutrient availability, particularly across underrepresented regions. Improved standardisation of experimental designs and more comprehensive reporting of nutritional outcomes will strengthen future evidence synthesis. From a policy and agricultural perspective, targeted breeding programmes, biofortification approaches, and precision nutrient management strategies should be advanced to maintain nutritional quality alongside productivity. Such coordinated efforts will be essential to ensure that rising atmospheric CO_2_ does not undermine the nutritional contribution of widely consumed leafy vegetables in future food systems.

## Data Availability

All data generated or analysed during this study are included in this published article and its Supplementary Information File.

## Supplementary Materials

The following supporting information can be downloaded at: https://www.mdpi.com/article/10.3390/biology15020152/s1, Word docx file, File S1: “S1 Supplementary Material File”; Excel datasheet, File S2: “S2_Data”; RMD script, File S3: “S3 Meta-analysis script”.

## Abbreviations

The following abbreviations are used in this manuscript:

CO_2_	Atmospheric carbon dioxide
eCO_2_	Elevated carbon dioxide
RuBisCO	The enzyme ribulose-1,5-bisphosphate carboxylase/oxygenase
FACE	Free-air CO_2_ enrichment
ppm	Parts per million
CEE	Collaboration for Environmental Evidence guidelines
PECO	Population, Exposure, Comparator and Outcome eligibility criteria
