# Supplementary material for: Effects of Elevated CO2 on Yield and Nutritional Quality of Kale and Spinach: A Meta-Analysis
Source: Biology (Basel). 2026 Jan 15;15(2):152. doi: 10.3390/biology15020152 (PMC12837620; doi:10.3390/biology15020152)
Supplement: Supplementary file 1 [file biology-15-00152-s001.zip › S1 Supplementary Material File.pdf]

Table of contents:

Supplementary Methods: Full methodology – literature search, study selection and statistical analyses	p2
Supplementary Results: Results and R scripts	p8

Supplementary Methods: Full methodology – literature search, study selection and statistical analyses

We employed a methodical approach in our meta-analysis. The formulation of our research questions and the identification and selection of relevant material adhered strictly to the guidelines set out by the Collaboration for Environmental Evidence standards and Standards for Evidence Synthesis. We began by utilising the research question outlined with a systematic map, "What published evidence exists for the effects of elevated carbon dioxide on the nutritional components of kale and spinach crops?" to define our PECO components and narrow down our primary and secondary questions. Selecting papers was done based on evidence clusters identified in the systematic map (more on this later). Here, we provide an in-depth description of our methods, including how we selected the papers and obtained the data for analysis.

Review questions and PECO components

.

The following primary question drove this meta-analysis:

1. "What are the effects of elevated carbon dioxide on the nutritional constituents of kale and spinach crops?"

Secondary questions included:

2. "How do the overall effects differ between the crop types (kale and spinach)?"
3. "How do CO_2 effects differ between the outcomes or constituent categories (carbohydrate, mineral, vitamin, nitrogenous compounds, yields, photosynthetic parameters and other group-classed phytochemicals)?"
4. "How do CO_2 effects differ between CO_2 levels (650-800ppm, 900-1000ppm, 1300-1900ppm, 3000ppm and above)?"
5. "How do CO_2 effects differ between the number of days of exposure?"
6. "Within kale crops, how do CO_2 effects differ between species types?"
7. "How do CO_2 effects differ between the outcomes or constituent categories

within kale crops?"

8. "How do CO_2 effects differ between species types
9. within spinach crops?"
10. "How do CO_2 effects differ between the outcomes or constituent categories within spinach crops?"

Overall, our research question can be summarised as:

"What are the effects of **elevated carbon dioxide levels** on the nutritional constituents and related parameters of **kale** and **spinach** crops, and how do these effects vary based on **crop type**, **CO_2 concentration** (650-800ppm, 900-1000ppm, 1300-1900ppm, 3000ppm and above), **duration of exposure**, **species type**, and **specific outcome categories** (carbohydrate, mineral, vitamin, nitrogenous compounds, yields, photosynthetic parameters and other group-classed phytochemicals)?"

With an emphasis on research clusters, Supplementary Table S1 has a list of the PECO components chosen for this work.

Supplementary Table S1: List of PECO components selected for the meta-analysis

PECO Component	Inclusion Criteria	Exclusion Criteria
**Population (P):**	Studies focusing specifically on kale (all species besides *Brassica oleracea*) and spinach (all species besides *Spinacia oleracea*) crops, including various cultivars.<br><br>Level 2 - **Kale** and **Spinach**<br><br>During data extraction, studies were clearly categorised as kale or spinach. The species and cultivar names were also categorised and classed to allow for sub-group analyses by crop, species and cultivar type.	Studies examine all taxonomic groups, crops, or population categories other than kale and spinach or where kale and spinach are not the primary subjects. Studies that combine kale and spinach data with other crops without disaggregating the results for kale and spinach alone.
**Exposure (E):**	Experimental studies where kale and spinach crops are exposed to elevated CO_2 levels, with CO_2 concentrations ranging from 650 ppm to levels exceeding 3000 ppm.<br><br>During extraction, point data from studies were clearly categorised as 650-800ppm, 900-1000ppm, 1300-1900ppm, or 3000ppm and above to allow for sub-group analyses by CO_2 levels.	Studies that do not focus on the specified CO_2 ranges or those where CO_2 exposure is combined with other factors, such as drought, heat, or nutrient stress, without providing standalone CO_2 data.
**Comparator and Study Design (C):**	Studies compare crops grown under elevated CO_2 conditions with those grown under ambient or lower CO_2 levels (typically around 400-450 ppm). Experimental designs that include a clear control group or baseline comparator.<br><br>The exposure period was also noted during data extraction to allow for sub-group analyses.	Studies lacking a control group or baseline comparator, observational studies without experimental control, or studies that combine CO_2 effects with other environmental factors without separating the CO_2-specific data.
**Outcomes (O):**	Primary outcomes include changes in the nutritional constituents of kale and spinach, specifically carbohydrates, minerals, vitamins, nitrogenous compounds, yields, photosynthetic parameters, and other group-classed phytochemicals.<br><br>During extraction, point data from studies were clearly categorised, as were specific parameter names (such as plant height, width, etc.). Units were placed without changes to allow for sub-group analyses by outcome categories.	Studies that do not report on relevant outcomes or those where outcomes are influenced by multiple factors (e.g., CO_2 and drought) without isolating the effects of CO_2 alone. Studies that aggregate data for multiple crops without providing separate outcomes for kale and spinach.

Literature Search, Study Selection, and Data Extraction

Several academic databases and search engines were used in a methodical manner to ensure that all pertinent papers were covered thoroughly throughout the literature search for this meta-analysis. The search aimed to locate peer-reviewed literature that looked at how the nutritional components of spinach and kale crops were affected by high CO_2 levels. Scopus, ScienceDirect, Google Scholar, CAB Abstract, PubMed, and Web of Science Core Collection were among the databases that were searched. The previous update was made on March 12, 2024, and all searches were done in English.

The reference management programme Zotero was used to import the articles found through these searches into a single library. This method made it easier to organise and handle the literature efficiently throughout the research selection and data extraction processes. A two-step hierarchical methodology served as the basis for the study selection process, guaranteeing that only highly relevant and methodologically rigorous research was included in the final analysis.

An initial assessment of the study's title and abstract was the first step in the selection process. This first step aimed to weed out irrelevant studies, like those that focused on crops other than spinach and kale, that did not look into eCO_2 exposure or combined CO_2 with other environmental factors (like heat or drought) without providing standalone CO_2 data. Following this first screening, articles were put through a full-text review. A predetermined set of qualifying criteria aligned with the PECO (Population, Exposure, Comparator, Outcome) framework was the basis for the full-text evaluation. Studies were included if they specifically examined kale or spinach crops, exposed these crops to elevated CO_2 levels within the specified range (650 ppm to >3000 ppm), and provided a clear comparator group (e.g., ambient CO_2 conditions). Studies were excluded if they lacked a proper control, combined CO_2 effects with other factors without disaggregated data, or failed to report relevant outcomes. Each study's meta-data, including study population, CO_2 exposure levels, comparator conditions, and outcomes, were systematically extracted and coded following a standardised framework to ensure consistency and accuracy.

From the initial pool of 872 articles identified through the systematic map, 138 duplicate entries were identified and removed using Zotero's duplicate detection feature, leaving a final set of 734 unique articles. Each of these articles was assigned a unique identifier and subjected to a PECO selection filter via the SysRev analytical tool, which enabled a more refined, transparent and cooperative screening process where all titles and abstracts were screened. Following the application of the PECO filters, 42 articles were selected for a detailed quality and validity assessment with full-text retrieved (7 full texts could not be assessed). The quality and validity assessment focused on determining whether the studies provided sufficient statistical information, such as means, variances, and sample sizes, necessary for inclusion in the meta-analysis. Another 29 studies were excluded because they were either having incomplete data, having combined data on effects of eCO_2 and other environmental stressors which could not be isolated or lacking necessary statistical details such as means, variance, standard deviation/error, etc. which could also not be obtained from authors (and co-authors) directly. Ultimately, 13 studies were retained, which contributed to a total of 346 effect sizes. The review was done in tandem by 2 reviewers, and any disputes were passed to a 3rd reviewer.

Data extraction was carried out from the chosen studies' text, tables, and figures. Precise numerical values were extracted from graphical data using the program metaDigitize (R Studio version 4.3.3). When raw data were available, summary statistics were computed immediately. During the data extraction phase, an outlier analysis was carried out, which led to the inclusion of 339 effect sizes in the final meta-analysis. Additionally, data was retrieved and categorised according to possible modifiers, including species type, length of CO_2 exposure, and particular outcome categories. Supplementary Figure S1 provides a graphic summary of the literature search, study selection, and data extraction procedure. This rigorous approach ensured that the final dataset used in the meta-analysis was both comprehensive and of high quality, providing robust insights into the effects of elevated CO_2 on kale and spinach crops.

ROSES Flow Diagram

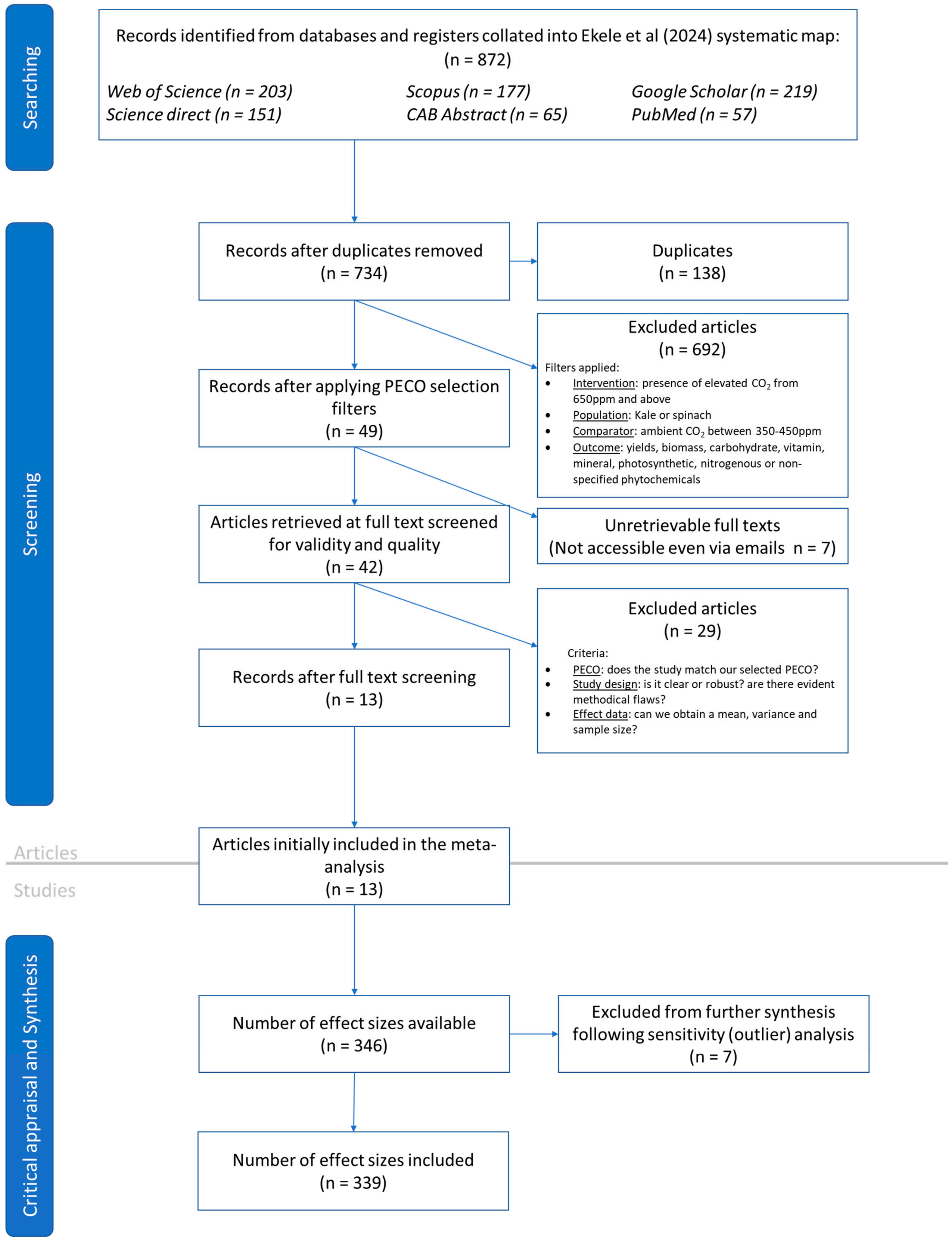

Supplementary Figure S1: Flow diagram illustrating the study selection process

Effect size and variance calculations

The influence of increased CO_2 on the nutritional components of spinach and kale crops was assessed in our meta-analysis primarily through effect size and variance estimations. For this study, 346 effect sizes were retrieved from 13 papers that included the data needed to compute one or more effect sizes. Here are the procedures for determining effect sizes and the variations that go along with them.

The difference between the CO_2-exposed crops and the control groups was measured using effect sizes. We utilised Hedges' g, a commonly used effect size metric that is especially appropriate for our meta-analysis as it accounts for small sample sizes. The standardised mean difference, or Hedges' g, is computed as follows:

$$g = \frac{M_1 - M_2}{SD_{pooled}}$$

Where M_1 and M_2 represent the means of the CO_2-exposed and control groups, respectively, and SD_{pooled} is the pooled standard deviation. The pooled standard deviation is calculated using:

$$\mathrm{SD}_{pooled} = \sqrt{\frac{(n_1 - 1).s_1^2 + (n_2 - 1).s_2^2}{n_1 + n_2 - 2}}$$

Here, s_1 and s_2 denote the standard deviations of the CO2-exposed and control groups, and n_1 and n_2 are their respective sample sizes.

To adjust for small sample sizes, we applied Hedges' correction factor J, given by:

$$J = 1 - \frac{3}{4(n_1 + n_2) - 9}$$

Thus, the corrected effect size is:

$$g_{corrected} = g.J$$

The variance of each effect size was calculated to estimate the precision of the effect size estimates. The standard error of the effect size g is calculated using:

$$\mathrm{SE}_{(g)} = \sqrt{\frac{J^2(\frac{1}{n_1} + \frac{1}{n_2}) + \frac{g^2}{2(n_1 + n_2)}}{n_1 + n_2 - 2}}$$

Where n_1 and n_2 are the sample sizes for the CO2-exposed and control groups, respectively.

To ensure that all accessible data were included in the meta-analysis, metaDigitize software (R Studio version 4.3.3) was used to extract data from figures and transform graphical data into numerical form. Accurate effect size estimations from trials using graphically represented data required this procedure.

Supplementary File 2 contains the dataset, which includes thorough effect size calculations and variance estimations.

Statistical Analysis

In order to thoroughly assess the impact of increased CO_2 on the nutritious components of spinach and kale crops, a statistical analysis was conducted for this meta-analysis. The effect sizes that were taken out of the 13 studies that satisfied our inclusion requirements were combined using a number of meta-analyses. The {metafor} package, version 3.8.1, was used in conjunction with the R environment (R version 4.3.3) for all statistical analyses. Supplementary Files 3 and 4 provide the R scripts and dataset utilised in these studies.

In order to evaluate the overall effect of increasing CO_2 on the nutritional quality of kale and spinach, we first built an overall meta-analytic model incorporating all known effect sizes ($n = 339$ after outlier elimination). Data on a variety of nutritional components, such as carbohydrates, minerals, vitamins, nitrogenous compounds, yields, and photosynthetic characteristics, were included in this model. The entire model sought to determine if, on average, increased CO_2 had a substantial impact on these nutritional characteristics for both crop types and to reflect the central trend of the effect sizes. The genuine effects may range from research to study owing to variations in experimental settings, CO_2 exposure levels, and crop species. To account for this variability, the model was fitted as a random-effects model. The Q-test and I^2 statistic were used to evaluate the heterogeneity across effect sizes; considering the variety of the included studies, significant heterogeneity was anticipated.

We performed a single-moderator analysis after the overall model to investigate how different aspects influenced the observed effects. These moderators included:

- Crop Type (Kale vs. Spinach): To examine whether the effects of eCO_2 differed between kale and spinach.
- Constituent Category: To determine if the impact of eCO_2 varied across different nutritional components (e.g., carbohydrates, minerals, vitamins).
- CO2 Exposure Level: To assess whether the magnitude of eCO_2 exposure (categorised into 650-800 ppm, 900-1000 ppm, 1300-1900 ppm, and 3000 ppm and above) influenced the effect sizes.
- Duration of Exposure: To investigate how the length of eCO_2 exposure impacted the nutritional outcomes.
- Species Type (Within Kale and Spinach): To evaluate whether different species within the kale and spinach categories responded differently to eCO_2.

We created multi-moderator models in addition to single-moderator models to take into consideration the combined effect of several elements. We were able to evaluate the moderator interaction and determine which moderators were most important in contributing to the variety of effect sizes thanks to these models. To guarantee the best match, the models' complexity was compared to the Akaike Information Criterion (AIC). To find and exclude extreme effect sizes that could disproportionately affect the final outcomes, we performed an outlier analysis. Outliers were found using standard diagnostic methods, such as looking at residual plots and calculating Cook's distance. Subsequently, a sensitivity study was conducted to evaluate the robustness of the results. This involved rerunning the meta-analyses both with and without the discovered outliers. We visually examined funnel plots for asymmetry, which might suggest a bias towards publishing important findings, in order to assess the possibility of publication bias. Because of the structure of our random-effects model, we were unable to use Egger's regression; nonetheless, the funnel plots' apparent asymmetry indicated that the meta-

analysis's publication bias was negligible. The {orchaRd} package was utilised to visualise the outcomes of our models. It offered easily comprehensible images that showed the impact sizes and associated confidence intervals.

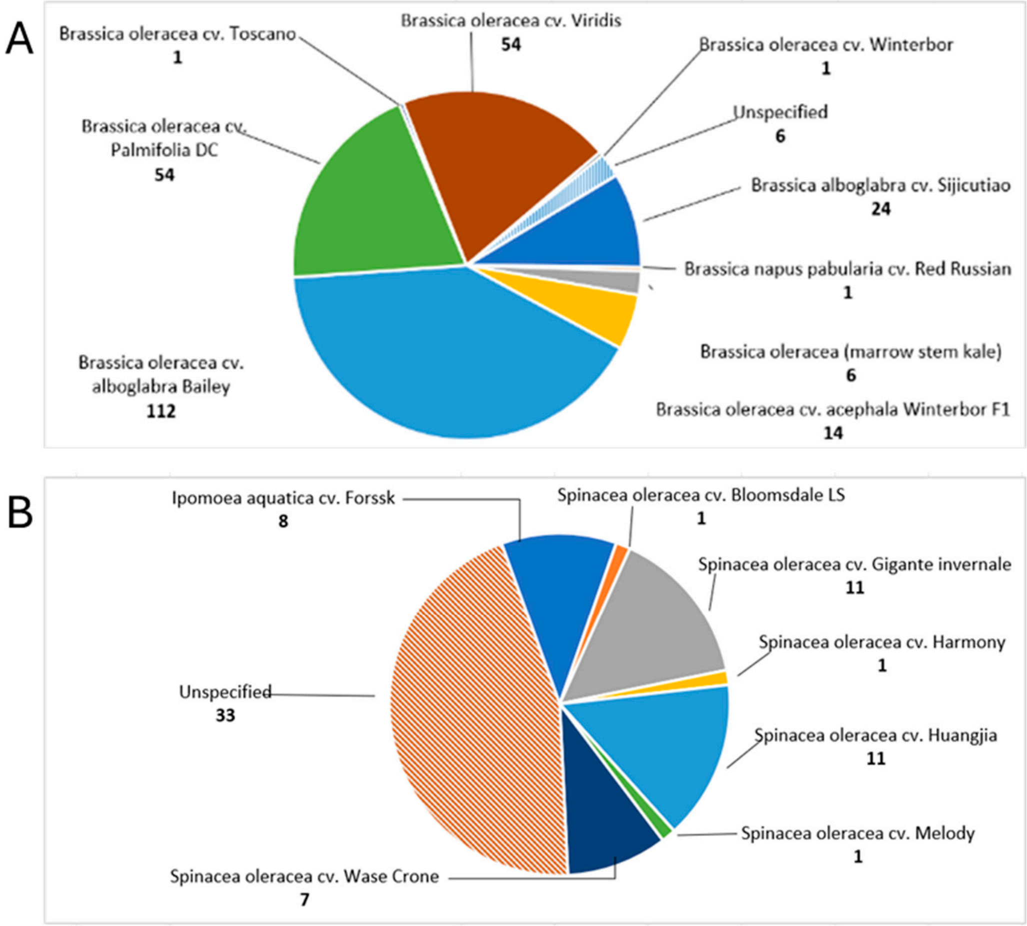

Supplementary Figure S2: Effect size distribution per species type. A for kale and B for spinach

Supplementary Results: Figures

For code, please see supplementary RMD file

Outlier identification and sensitivity analysis

Overall model (all data)

All outliers

```
#Identifying all outliers
dat$upperci<-dat$Hedgesg + 1.96*dat$HedgesSE
dat$lowerci<-dat$Hedgesg - 1.96*dat$HedgesSE
dat$outlier<-dat$upperci<mod0$ci.lb | dat$lowerci>mod0$ci.ub
sum(dat$outlier) #84 potential ES outliers!
dat[dat$outlier, c("StudyID", "ESID", "Hedgesg", "upperci", "lowerci")]
ggplot(data = dat, aes(x = Hedgesg, colour = outlier, fill = outlier)) +
  geom_histogram(alpha = .2) +
  geom_vline(xintercept = mod0$b[1]) +
  theme_bw()
```

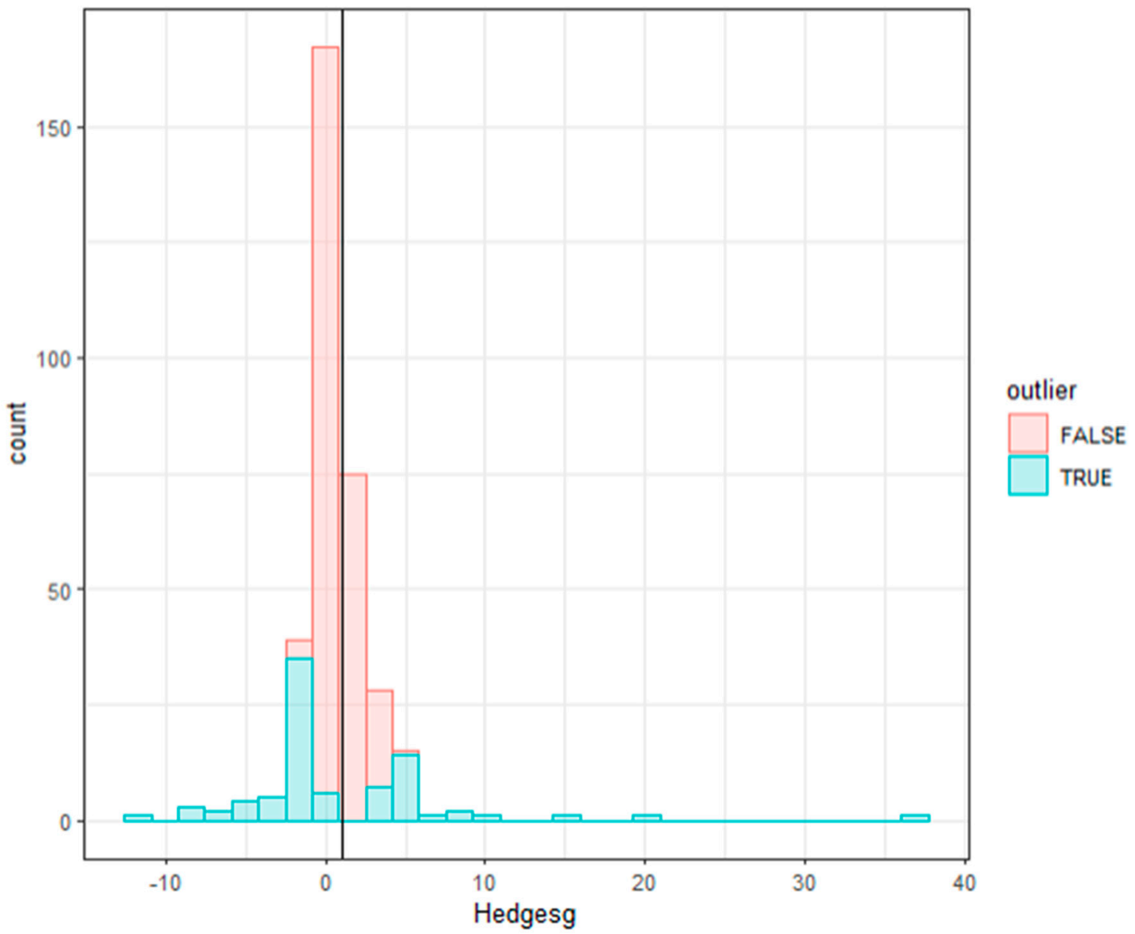

Supplementary Figure S3: Effect size histogram displaying the results of the outlier analysis. The effect sizes that are not regarded as outliers are shown in red, while those that are shown in blue. Since the majority of the outliers are consistently distributed between -10 and +10, they do not represent outliers. However, there is some remaining asymmetry with effect sizes >+10. These are likely true outliers (for g>10). Looking at our dataset, g>10 only stems from seven effect sizes: ESID 143, 152, 154, 197, 221, 260, 315.

Removing effect sizes: ESID 143, 152, 154, 197, 221, 260, 315

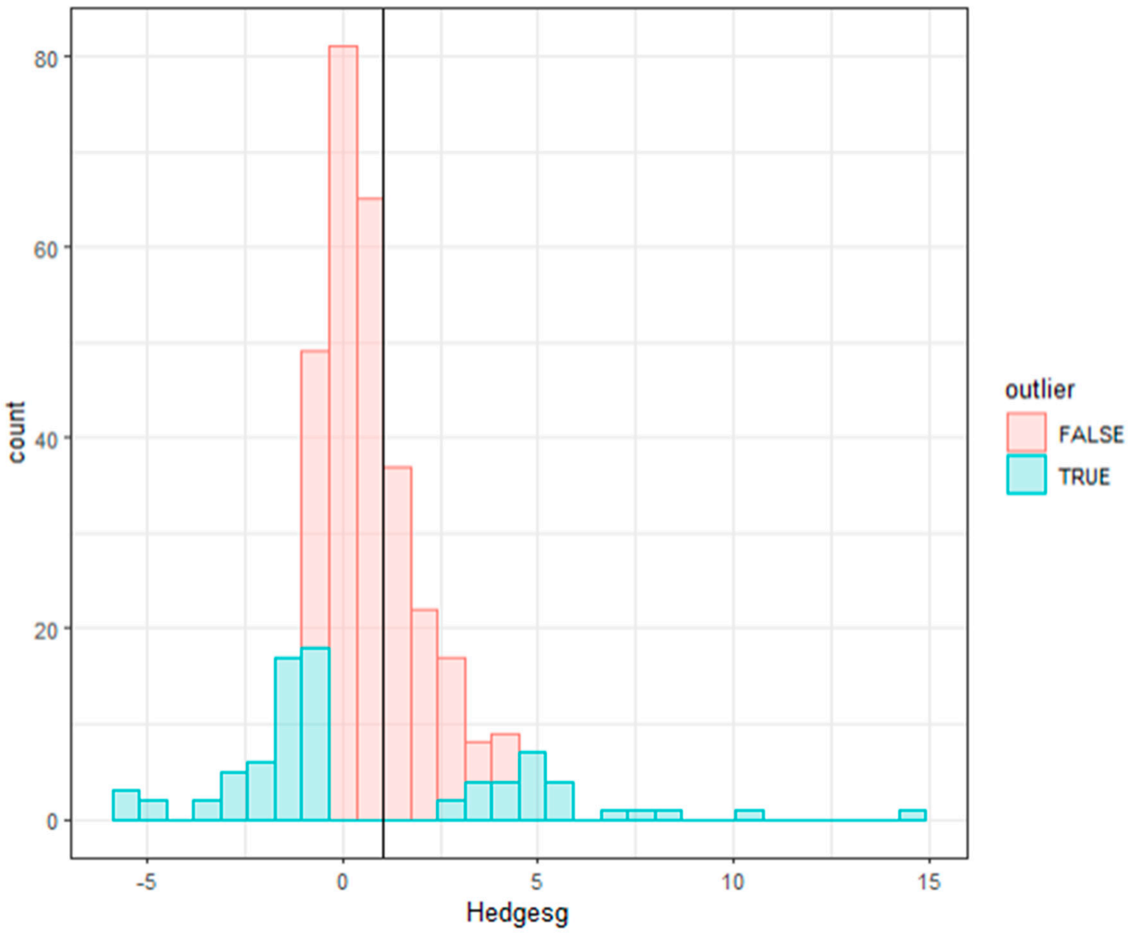

Supplementary Figure S4: The effect size histogram, which includes 339 effect sizes from all 13 papers, displays the results of the outlier analysis. It is generally symmetrical. There is no proof that these outliers skewed the pooled effect. → We can keep them and remove ESID 143,152,154,197,221,260,315 only.

Overall model

Overall, the model shows significant moderate (~1.04) positive effects of eCO_2 on crops compared to ambient CO_2 levels. Based on 339 effect sizes from 13 studies (pval<0.01 *** (p=0.0043)).

Supplementary Table S2: Model estimates, Confidence Interval, Prediction Interval, and I2 heterogeneity values for the overall meta-analytical model. Significant effect sizes are shown in bold.

Overall model	estimate	lowerCI	upperCI	lowerPR	upperPR	I2_Total	I2_StudyID	StudyID/ESID
	1.0406	0.3951	1.6861	-2.3245	4.4058	85.4202	35.6089	49.8113

Large residual heterogeneity is largely due to between-study (35.6%) and within-study (49.8%) heterogeneity.

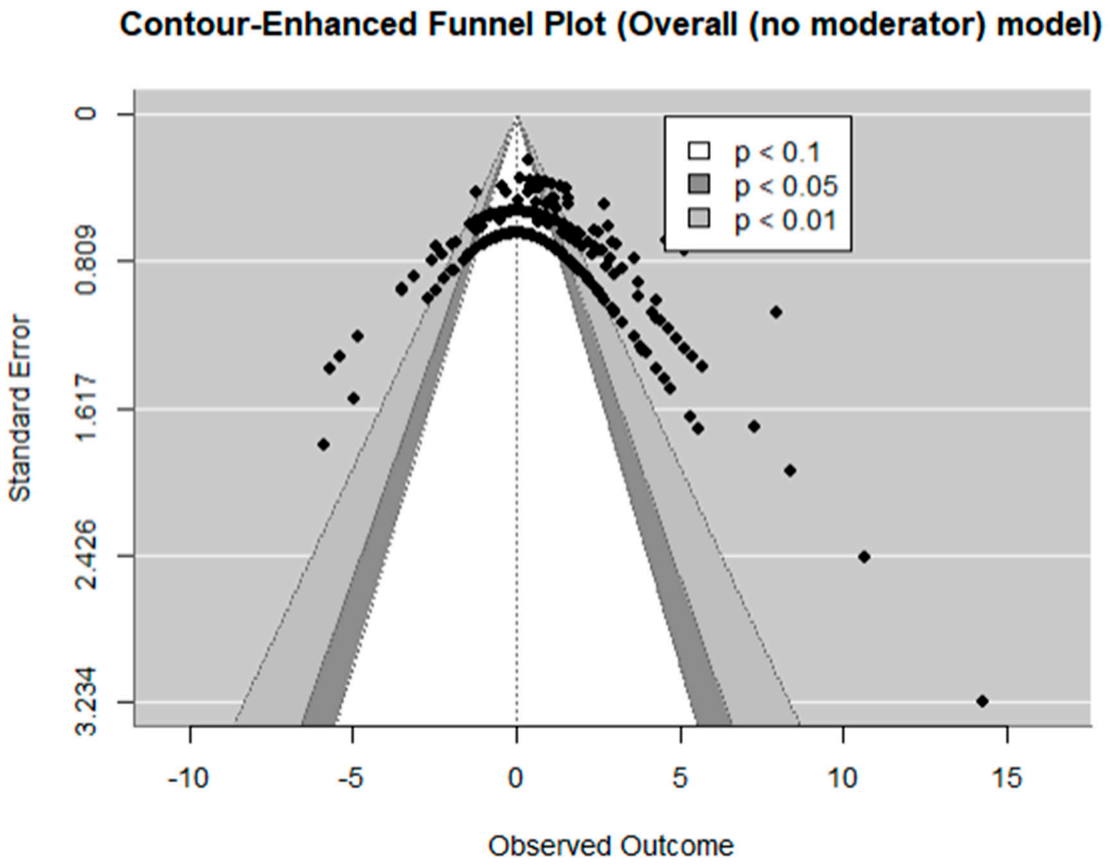

Supplementary Figure S5: Funnel plot for the overall (simple, no moderator) model for the effects of eCO_2 on kale and spinach crops. Evidence of asymmetry, therefore, is a sign of publication bias.

Single moderators' models

By Crop type (common name: kale or spinach)

The model shows significant positive responses of kale (Hedges g=1.0340; p=0.0089) and spinach to eCO_2 (slightly higher Hedges g=1.0608; p=0.9634). The Omnibus Test of Moderators: QM(df1 = 2, df2 = 337) = 5.703, *P*=0.0037.

Supplementary Table S3: Model estimates, Confidence Interval, Prediction Interval, p-values and I^2 heterogeneity values for the meta-analytical model using seabed type as moderator. Significant effect sizes are shown in bold

Moderator level	Effect size estimate	Lower CI	Upper CI	Lower PR	Upper PR	p-value	I2_Total	I2_StudyID	StudyID/ESID
Kale	1.03401	0.1694	1.8987	-2.4850	4.5530	0.0089**	85.9642	37.9688	47.9954
Spinach	1.06079	0.0468	2.0748	-2.4979	4.6194	0.9634			

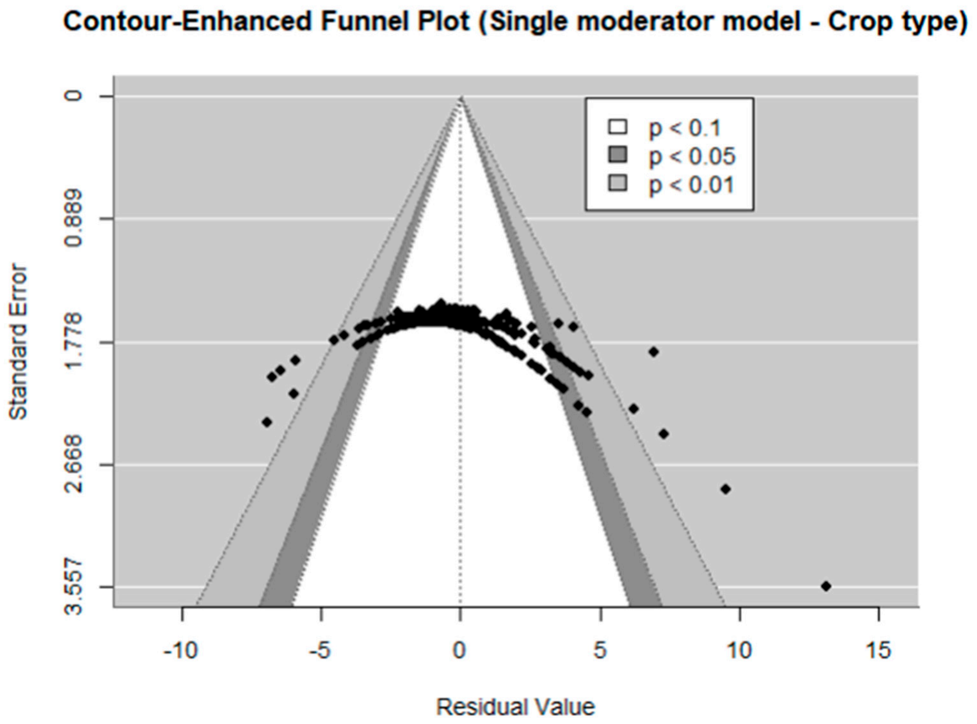

Supplementary Figure S6: Funnel plot for the single moderator model using crop type as moderator for the effects of eCO_2 on spinach and kale. There is no evidence of asymmetry and, therefore, no sign of publication bias.

By Outcome or constituent category

When looking at effects by outcome or constituent category, it appears that effects vary largely. While significant large positive effects are obvious for carbohydrates, nitrogenous compounds, photosynthetic, and yield, other categories have non-significant effects, such as minerals, vitamins, and other phytochemicals. The Omnibus Test of Moderators detected significant differences between outcome or constituent category (QM(df1 = 7, df2 = 332) = 4.0457, p-val = 0.0003), and pairwise contrasts revealed that carbohydrates and mineral contents behaved significantly differently to all other categories

Supplementary Table S4: Model estimates, Confidence Interval, Prediction Interval, p-values and I^2 heterogeneity values for the meta-analytical model using outcome or constituent category as moderator. Significant effect sizes are shown in bold

Moderator level	Effect size estimate	Lower CI	Upper CI	Lower PR	Upper PR	p-value	I2_Total	I2_StudyID	StudyID/ESID
Carbohydrate contents	1.33705	0.2182	2.4559	-2.4749	5.1490	0.0037**	84.9743	34.1127	50.8616
Mineral contents	1.06079	-0.6135	1.2108	-3.4579	4.0551	0.0135*			
Nitrogenous compounds	1.21258	0.3845	2.0406	-2.5244	4.9495	0.7657			
Other phytochemicals	0.47249	-0.5694	1.5144	-3.3176	4.2626	0.0690			
Photosynthetic	1.17615	0.2820	2.0703	-2.5760	0.42457	0.7200			
Vitamin contents	0.42457	-0.6275	1.4767	-3.3683	4.2175	0.0516			
Yield	1.14611	0.4052	1.8870	-2.5725	4.8647	0.6264			

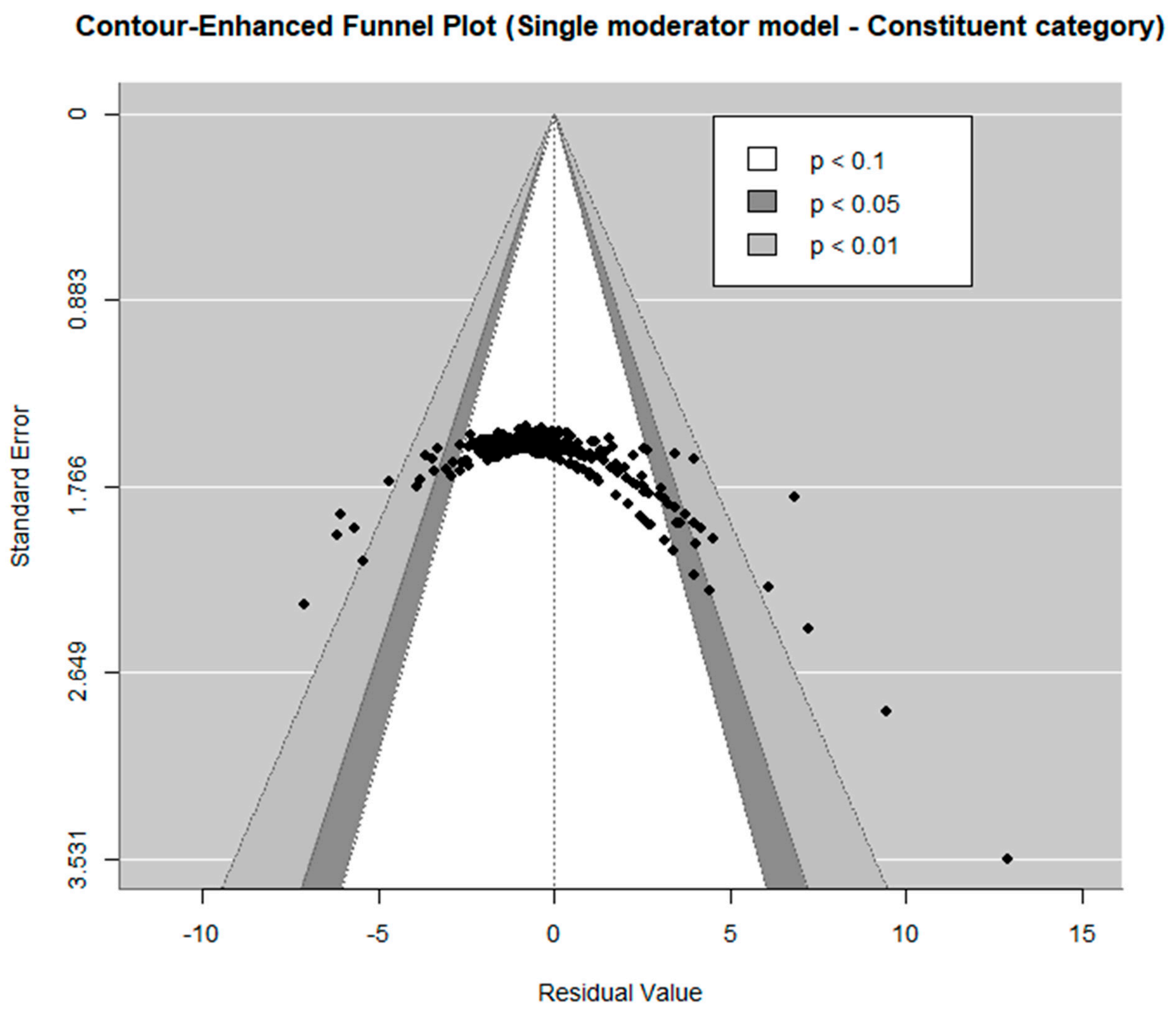

Supplementary Figure S7: Funnel plot for the single moderator model using outcome or constituent category as moderator for effects of eCO_2 on kale and spinach. There is no evidence of asymmetry and, therefore, no sign of publication bias.

By CO_2 levels

Now, we considered changes at different CO_2 level groupings, namely, "650-800ppm", "900-1000ppm", "1300-1900ppm", and "3000ppm-and-above". It appears that effects vary largely. (Omnibus Test of Moderators (QM(df1 = 4, df2 = 335) = 3.3138, p-val = 0.0111), and pairwise contrasts revealed that crops grown in 650-800ppm behaved significantly differently to all other levels, which had similar effects to one another.

Supplementary Table S5: Model estimates, Confidence Interval, Prediction Interval, p-values and I^2 heterogeneity values for the meta-analytical model using CO_2 levels as moderator. Significant effect sizes are shown in bold

Moderator level	Effect size estimate	Lower CI	Upper CI	Lower PR	Upper PR	p-value	I2_Total	I2_StudyID	StudyID/ESID
650-800ppm	0.95179	0.2132	1.6903	-2.6520	4.5555	0.0038**	86.11004	38.52279	47.58726
900-1000ppm	1.06175	0.1628	1.9607	-2.5783	4.7018	0.7292			
1300-1900ppm	1.19143	0.3459	2.0370	-2.4357	4.8186	0.3846			

3000ppm and above	1.47934	0.4970	2.4617	-2.1821	5.1409	0.1552			

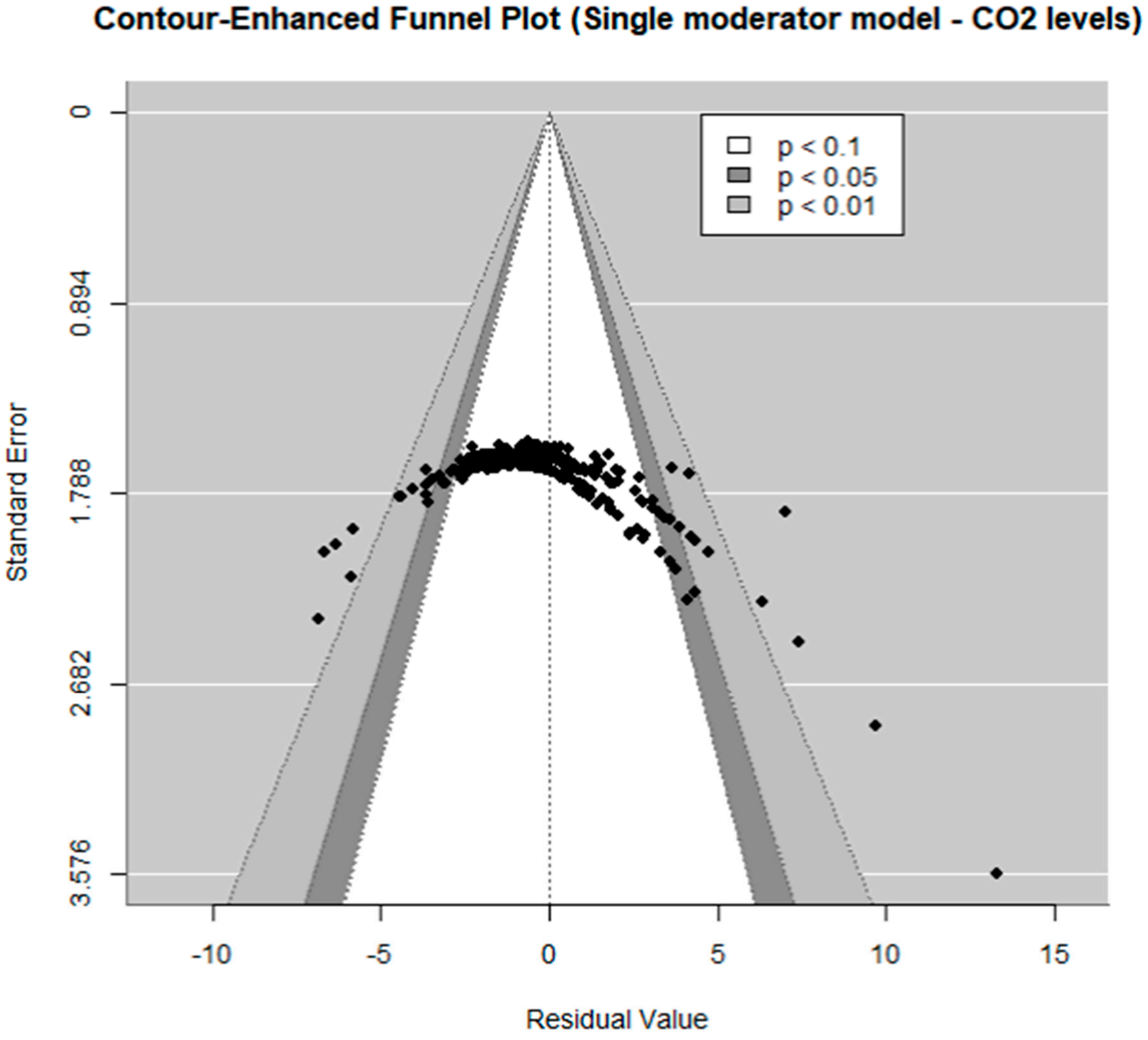

Supplementary Figure S8: Funnel plot for the single moderator model using CO_2 levels as moderator for effects of eCO_2 on kale and spinach. There is no evidence of asymmetry and, therefore, no sign of publication bias.

By Exposure Time (number of days)

The effect size estimates varied across number of days the crops were exposed to eCO_2 however, the Omnibus Test of Moderators detected no significant increase/decrease or difference categories (QM(df1 = 14, df2 = 1) = 0.5838, p-val = 0.7883) and pairwise contrasts revealed that they had similar effects to one another.

Supplementary Table S6: Model estimates, Confidence Interval, Prediction Interval, p-values and I^2 heterogeneity values for the meta-analytical model using exposure time category as moderator. Significant effect sizes are shown in bold

Moderator level	Effect size estimate	Lower CI	Upper CI	Lower PR	Upper PR	p-value	I2_Total	I2_StudyID	StudyID/ESID
14days	1.59830	-11.1610	14.3577	-27.2084	30.4050	0.3571	91.33067	60.90028	30.43038
16days	0.55803	-23.2956	24.4117	-34.5990	35.7151	0.6254			

20days	0.66543	-21.1256	22.4565	-33.1262	34.4571	0.7206			
25days	0.25405	-21.9142	22.4223	-33.7821	34.2902	0.6252			
26days	1.79967	-13.7956	17.3949	-28.3704	31.9698	0.9196			
28days	1.44649	-11.0590	13.9519	-27.2486	30.1416	0.5381			
29days	0.03693	-21.9939	22.0678	-33.9098	33.9837	0.5786			
30days	0.57603	-23.3146	24.4666	-34.6061	35.7582	0.6318			
35days	1.36199	-13.9065	16.6304	-28.6405	31.3645	0.9047			
40days	1.18554	-20.7436	23.1147	-32.6953	35.0664	0.8364			
43days	-0.2988	-24.1742	23.5767	-35.4707	34.8731	0.3739			
56days	1.07303	-21.0212	23.1673	-32.9149	35.0609	0.8371			
60days	0.72078	-21.1645	22.6061	-33.1317	34.5732	0.6601			
80days	0.74706	-21.1716	22.6658	-33.1270	34.6211	0.6701			

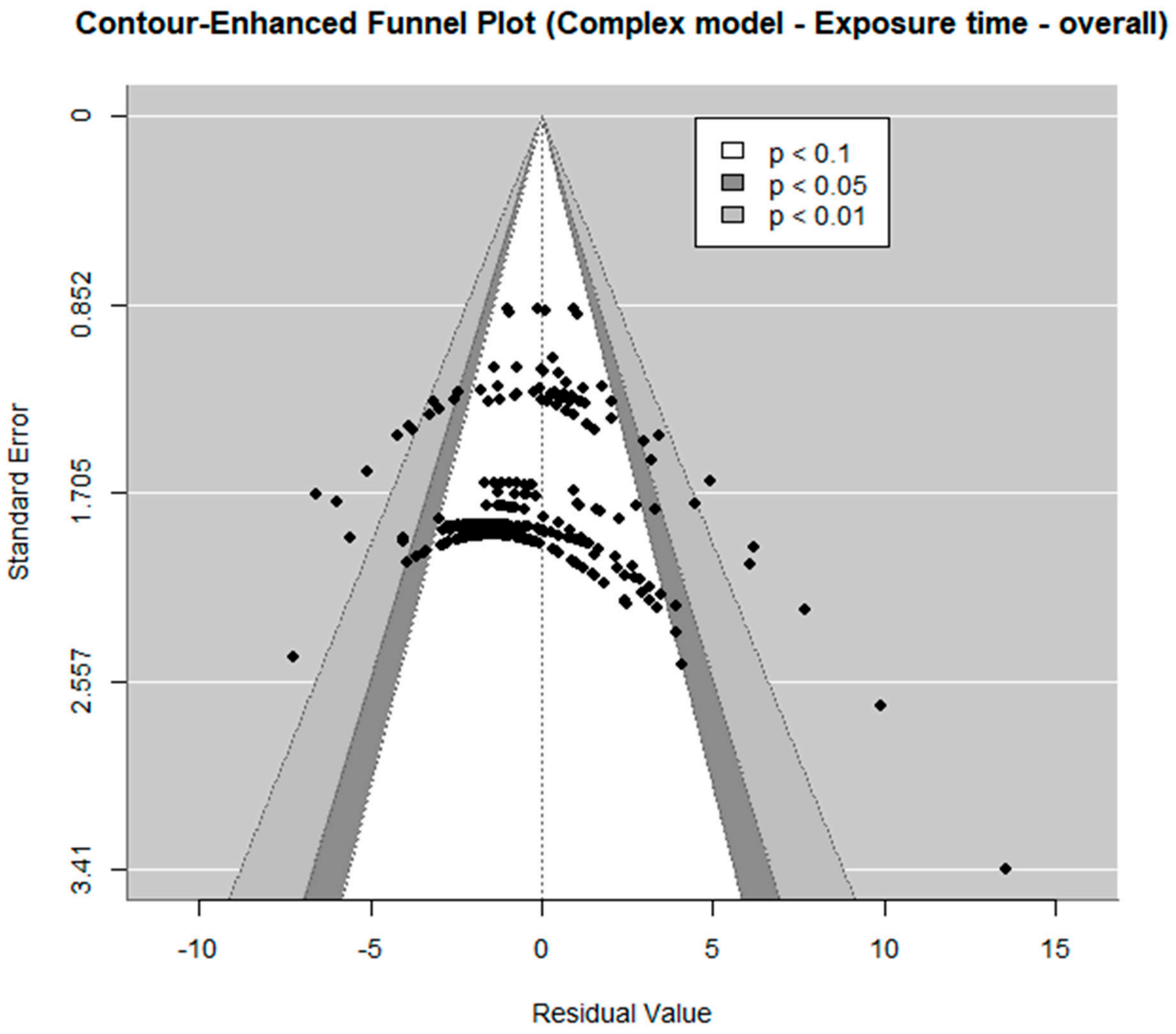

Supplementary Figure S9: Funnel plot for the single moderator model using exposure time category as moderator for effects of eCO_2 on kale and spinach. There is no evidence of asymmetry and, therefore, no sign of publication bias.

By Species category within each crop type

Kale

The effect size estimates varied across types of kale species that were exposed to eCO_2; however, the Omnibus Test of Moderators detected no significant effect or difference categories (QM (df1 = 10, df2 = 1) = 2.1289, p-val = 0.4913) and pairwise contrasts revealed that *Brassica oleracea* cv. Palmifolia DC and *Brassica oleracea* cv. Viridis behaved differently from the rest of the species, which had similar effects.

Supplementary Table S7: Model estimates, Confidence Interval, Prediction Interval, p-values and I^2 heterogeneity values for the meta-analytical model using species type category as moderator. Significant effect sizes are shown in bold

Moderator level	Effect size estimate	Lower CI	Upper CI	Lower PR	Upper PR	p-value	I2_Total	I2_StudyID	StudyID/ESID
Brassica oleracea cv. Winterbor	5.37030	-17.9947	28.735	-23.0262	33.7668	0.2100	81.25759	42.07943	39.17816
Brassica oleracea cv. Toscano	4.30562	-17.2064	25.8176	-22.5868	31.1978	0.6191			
Brassica napus pabularia cv. Red Russian	2.98162	-16.6368	22.6000	-22.4214	28.3847	0.2389			
Brassica oleracea (marrow stem kale)	0.27682	-12.3907	12.9443	-20.2390	20.7926	0.2481			
Brassica oleracea cv. acephala Winterbor F1	1.72283	-7.2384	10.6841	-16.7362	20.1819	0.3152			
Brassica alboglabra cv. Sijicutiao	0.17362	-11.1448	12.9886	-19.2285	21.0723	0.2772			
Brassica oleracea cv. alboglabra Bailey	0.17272	-11.5078	11.8551	-19.7485	20.0957	0.2398			
Brassica oleracea cv. Viridis	0.57603	-11.6199	11.9653	-19.8147	20.1602	**0.0122**			
Brassica oleracea cv. Palmifolia DC	0.36918	-11.4136	12.1520	-19.6125	20.3509	**0.0158**			
Brassica oleracea	1.05337	-11.6781	13.7849	-19.5020	21.6088	0.2875			

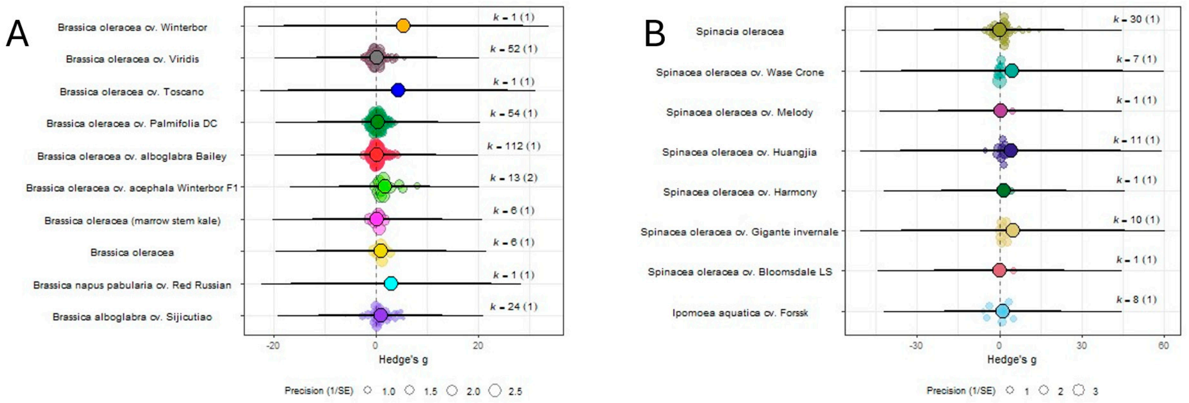

Supplementary Figure S10: A. Orchard plot showing the effect of eCO_2 on only kale crops with species type as a moderator. B. Orchard plot showing the effect of eCO2 on spinach crops only with species type as a moderator.

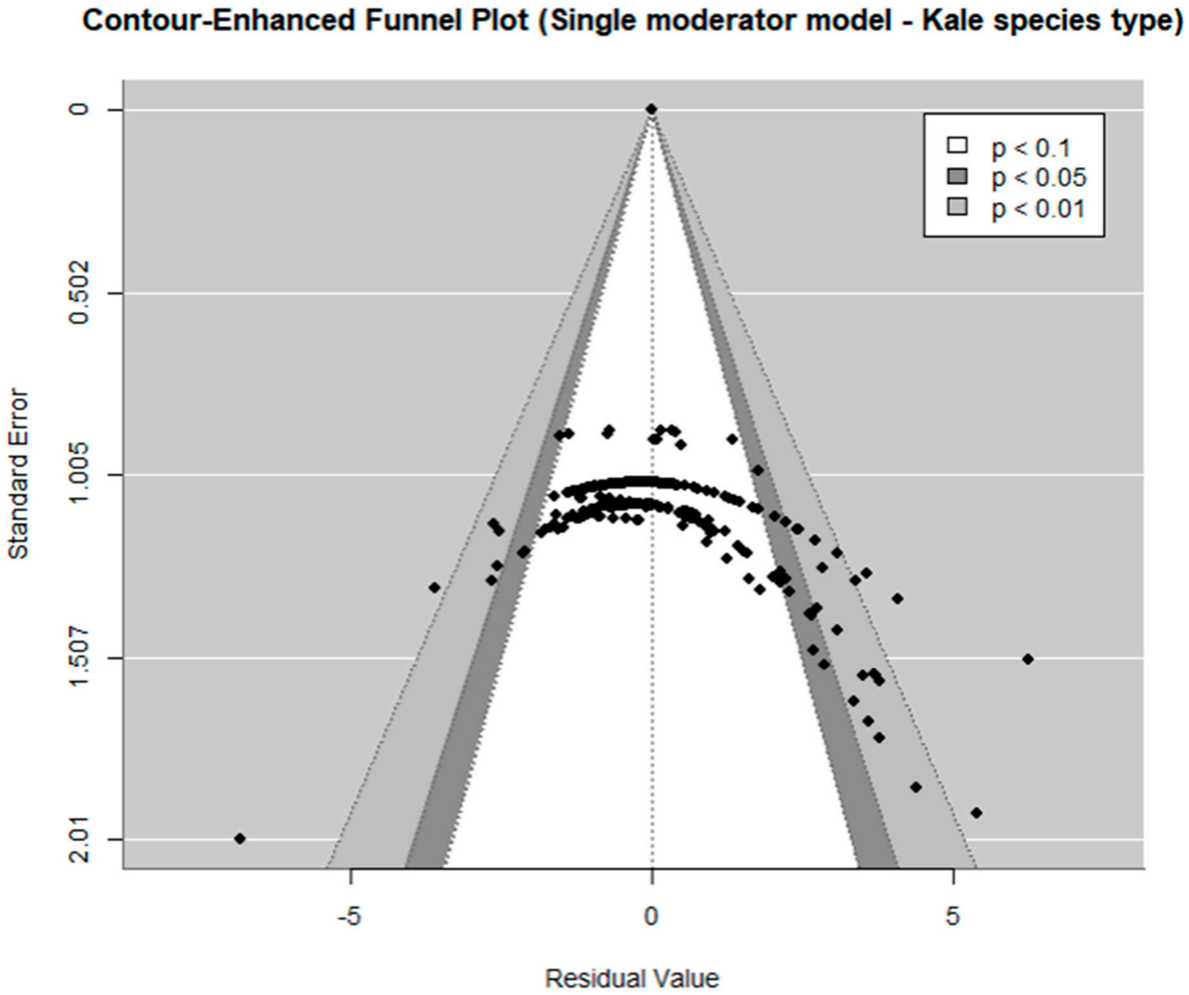

Supplementary Figure S11: Funnel plot for the single moderator model using species type category as moderator for effects of eCO_2 on kale. There is no evidence of asymmetry and, therefore, no sign of publication bias.

Spinach

Spinach had same results as kale (QM (df1 = 8, df2 = 1) = 0.7221, p-val = 0.7269).

Supplementary Table S8: Model estimates, Confidence Interval, Prediction Interval, p-values and I^2 heterogeneity values for the meta-analytical model using species type category as moderator. Significant effect sizes are shown in bold

Moderator level	Effect size estimate	Lower CI	Upper CI	Lower PR	Upper PR	p-value	I2_Total	I2_StudyID	StudyID/ESID
Ipomoea aquatica cv. Forssk	1.25046	-20.0310	22.5320	-42.0010	44.5020	0.5917	94.58353	27.61019	66.97334
Spinacea oleracea cv. Bloomsdale LS	0.05235	-23.6090	23.7137	-44.4184	44.5231	0.7159			
Spinacea oleracea cv. Gigante invernale	4.91046	-35.8954	45.7164	-50.6135	60.4344	0.3163			
Spinacea oleracea cv. Harmony	1.80126	-21.0063	24.6088	-42.2212	45.8237	0.8595			
Spinacea oleracea cv. Huangjia	4.15124	-35.9684	44.2709	-50.8704	59.1728	0.4202			
Spinacea oleracea cv. Melody	0.55501	-22.1549	23.2649	-43.4169	44.5269	0.8239			
Spinacea oleracea cv. Wase Crone	4.62357	-35.9109	45.1580	-50.7012	59.9484	0.3529			
Spinacia oleracea	0.02636	-23.7714	23.8241	-44.5172	44.5699	0.7114			

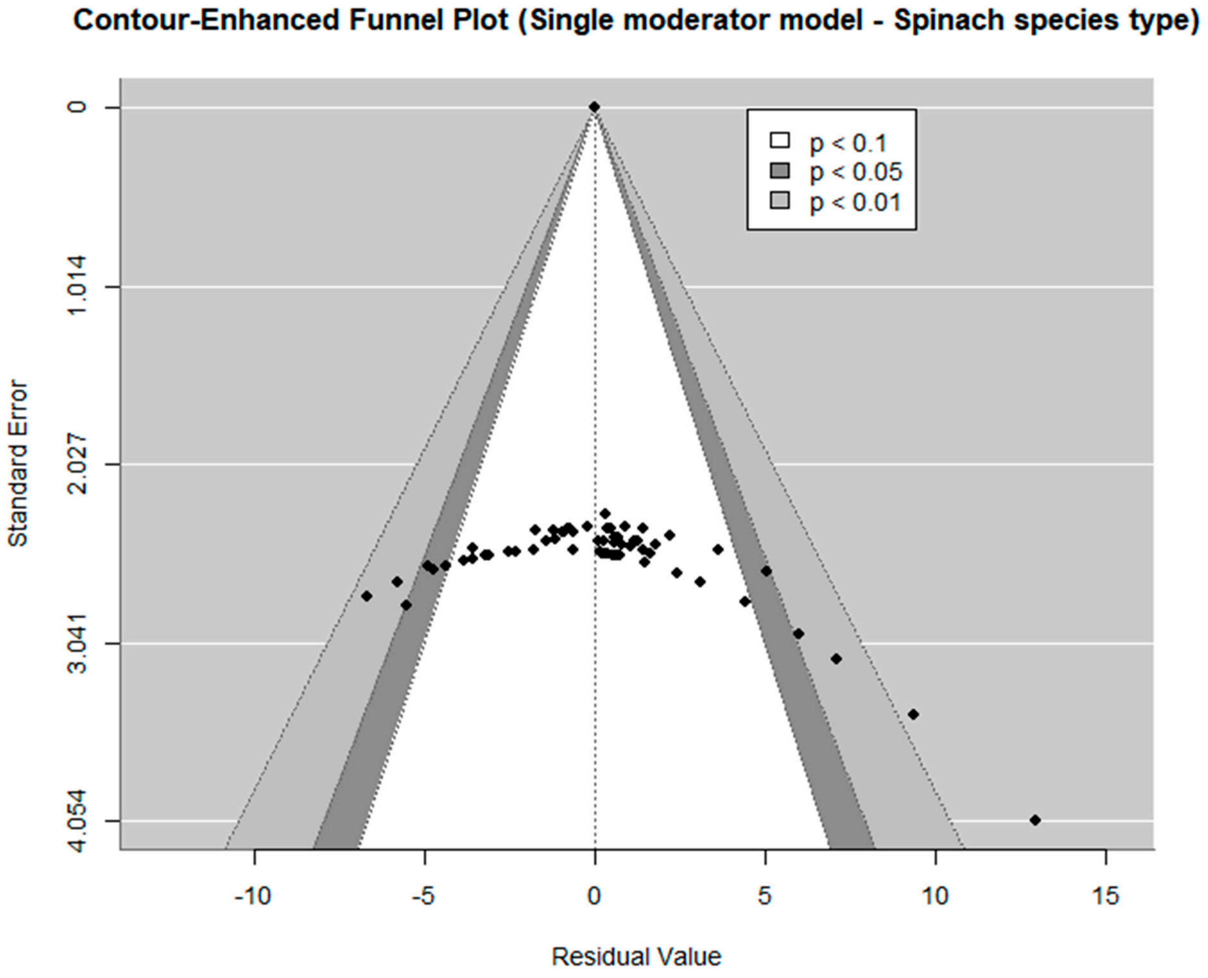

Supplementary Figure S12: Funnel plot for the single moderator model using species type category as moderator for effects of eCO_2 on spinach. There is no evidence of asymmetry and, therefore, no sign of publication bias.

Full (most complex) multi-moderator model

Significant effect of moderators (Test of Moderators (coefficients 1:15): QM (df1 = 31, df2 = 308) = 3.7943, p-val < .0001). The full model shows that the effects of eCO_2 on both kale and spinach varied across multiple moderators - crop type, category of outcome or constituent measured, and CO_2 level.

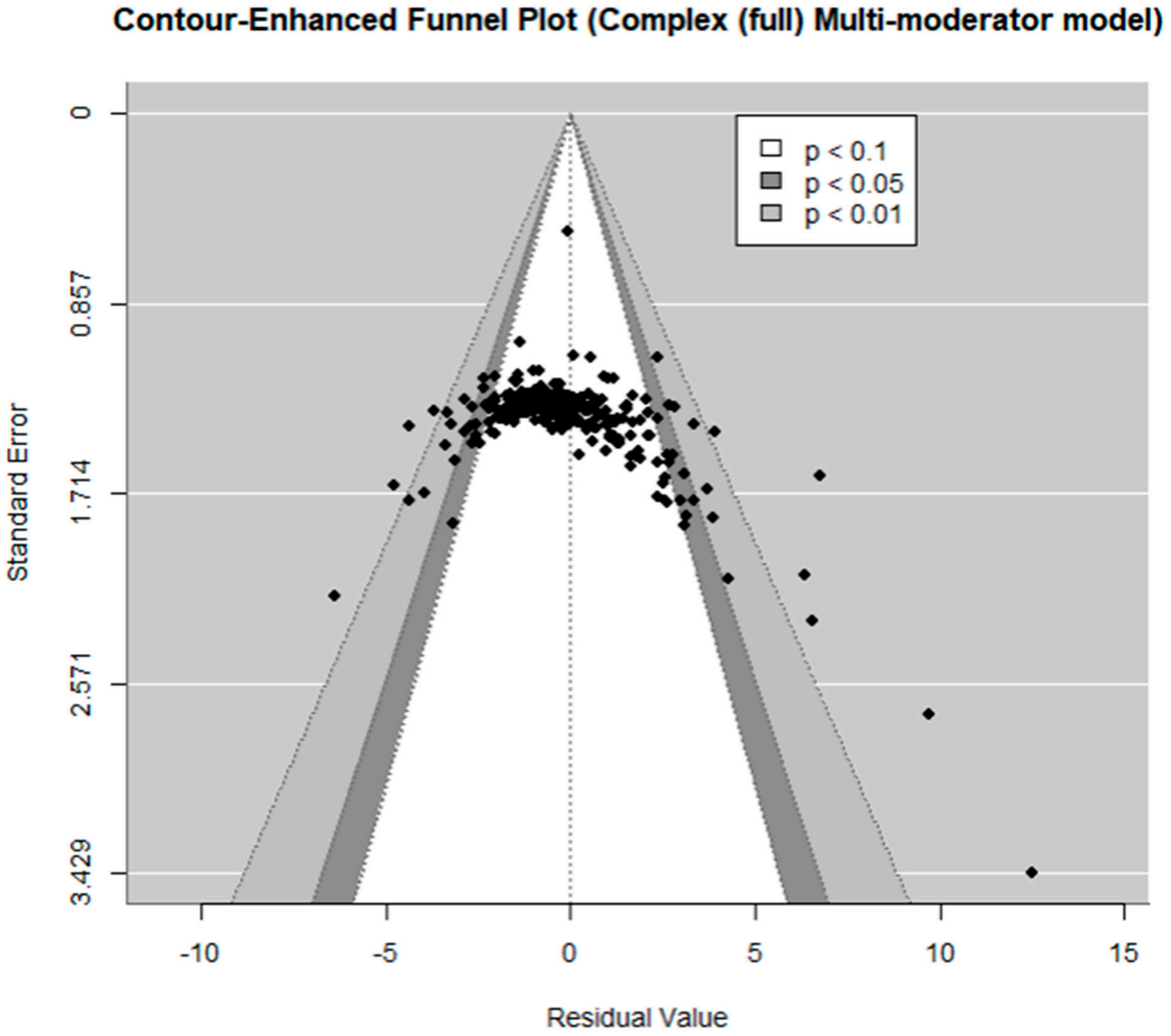

Supplementary Figure S13: Funnel plot for the complex (full) multi-moderator model for the effects of eCO_2 on both kale and spinach within various constituent/outcome categories and CO_2 levels. There is no evidence of asymmetry and, therefore, no sign of publication bias.

For visualisation and interpretation, additional plots were produced, and models were run for each constituent category of interest separately on sub-datasets split by crop type (kale and spinach). Models followed the same structures as the one used throughout and included one moderator-constituent measure.

Supplementary Table S9: Summary of the effect of eCO_2 across the different types of constituents measured in both kale and spinach. Significant effect sizes are shown in bold.

Moderator level - Crop type	Moderator level - Outcome category (group)	Moderator level - Type of constituent measured	Effect size estimate	Lower CI	Upper CI	Lower PR	Upper PR	p-value
Kale	Yield	Dry weight	0.05220	-0.6102	0.7125	-1.5206	1.6250	0.8773
		Fresh weight	2.15480	1.5156	2.7940	0.5916	3.7180	**<.0001**
		Leaf area	1.00770	0.3608	1.6546	-0.5587	2.5741	**0.0055**
		Leaf length	0.67031	-0.0637	1.4043	-0.9340	2.2746	0.1296

		Leaf number	-0.22831	-1.0027	0.5461	-1.8515	1.3949	0.3647
		Leaf weight	-0.07362	-0.7905	0.6433	-1.6702	1.5230	0.6650
		Leaf width	-0.24672	-1.0205	0.5271	-1.8696	1.3762	0.3334
		Plant height	0.95476	0.1663	1.7733	-0.6752	2.5847	**0.0046**
		Plant weight	-0.11694	-0.7571	0.5232	-1.6806	1.4467	0.5098
		Plant width	-0.01007	-0.7842	0.7641	-1.6332	1.6130	0.8405
		Root length	0.03353	-0.7394	0.8065	-1.5890	1.6560	0.9518
		Root surface area	7.22253	4.8831	9.5620	4.4824	9.9626	**<.0001**
		Root:shoot	1.97572	0.6030	3.3485	-0.0041	3.9555	0.0063
		Shoot diameter	0.57209	-0.4646	1.6087	-1.1913	2.3355	0.3485
Spinach	Yield	Biomass	0.3535692	-1.8203923	2.527531	-3.3029185	4.010057	0.7499
		Dry weight	5.1491793	1.2774858	9.020873	0.2877182	10.010640	**0.0343**
		Fresh weight	0.6799696	-0.9466902	2.306629	-2.6800625	4.040002	0.7366
		Leaf area	1.0033829	-0.8797023	2.886468	-2.4880070	4.494773	0.5265
		Leaf dry mass	2.2446604	0.5927373	3.896584	-1.1276746	5.616995	0.0569
		Shoot:root	0.9136354	-1.0545199	2.881791	-2.6243624	4.451633	0.6312
Kale	Carbohydrate contents	C:N	4.7170683	1.7563543	7.6777824	1.7557052	7.6784315	**0.0018**
		Carbohydrates	5.3095886	2.0434481	8.5757290	2.0428597	8.5763174	0.7922
		Carbon content	-0.1970417	-0.7458492	0.3517659	-0.7493404	0.3552571	**0.0014**
Spinach	Carbohydrate contents	C:N	0.8272584	-9.941353	11.595869	-14.34073	15.99525	0.8803
		Carbohydrates	4.7278168	-4.136279	13.591913	-9.15300	18.60863	0.5836
		Carbon content	2.4471025	-5.947720	10.841925	-11.13884	16.03305	0.8161
		Sucrose	2.0239350	-5.431031	9.478901	-11.00225	15.05012	0.8579

		Sugar	3.6149191	-7.180082	14.409920	-11.57182	18.80165	0.7201
Kale	Nitrogenous contents	4-hydroxyglucobrassicin	0.0800000	-2.10254988	2.26254988	-2.7281974	2.8881974	0.9427
		4-methoxyglucobrassicin	0.8001146	0.08325448	1.51697466	-1.1068037	2.7070328	0.5390
		Glucobrassicin	1.2646320	0.53656258	1.99270151	-0.6465283	3.1757924	0.3129
		N:S	-2.6762064	-5.33218410	-0.02022878	-5.8662952	0.5138823	0.1161
		Neoglucobrassicin	0.5168018	-0.18641496	1.22001852	-1.3850296	2.4186332	0.7089
		Nitrogen content	-5.9000000	-9.88809904	-1.91190096	-10.2620388	-1.5379612	**0.0099**
		Progoitrin	1.1183310	0.39812488	1.83853710	-0.7898476	3.0265096	0.3759
		Protein	0.9095974	-0.02525705	1.84445190	-1.0895028	2.9086976	0.4935
		Sinigrin	-0.6537911	-1.37596361	0.06838134	-2.5627128	1.2551305	0.5316
Spinach	Nitrogenous contents	Catalase	1.3726449	-2.909446	5.6547357	-4.495099	7.240389	0.5298
		Glutathione	2.2992375	-1.985979	6.5844544	-3.570788	8.169263	0.7643
		Nitrogen content	1.6261103	-2.684298	5.9365187	-4.262331	7.514551	0.9348
		Peroxidase	-0.7886585	-2.927239	1.3499219	-5.334826	3.757509	0.3761
		Proline	1.4750679	-2.739859	5.6899946	-4.343843	7.293979	0.9733
		Protein	-4.8472183	-9.522746	-0.1716911	-11.007954	1.313518	0.0545
Kale	Mineral contents	Ash	-1.60000000	-3.7242703	0.5242703	-4.1625201	0.9625201	0.1399

		Calcium	0.69838035	-0.3968796	1.7936403	-1.1053877	2.5021484	0.0595
		Iron	-0.23072365	-1.2808473	0.8194000	-2.0074466	1.5459993	0.2574
		Magnesium	-0.11327554	-1.3006030	1.0740519	-1.9743857	1.7478347	0.2312
		Phosphorous	0.06816957	-0.9812688	1.1176080	-1.7081484	1.8444876	0.1676
		Potassium	-0.46362089	-1.5165744	0.5893326	-2.2420179	1.3147761	0.3475
		Sulfur content	1.26990817	0.1751903	2.3646260	-0.5335307	3.0733471	**0.0186**
Spinach	Mineral contents	Cadmium	0.9718996	-0.91935409	2.8631532	-1.4194759	3.36327501	0.3138
		Calcium	-3.5001182	-5.89771446	-1.1025218	-6.3090867	-0.69114962	**0.0025**
		Iron	1.3174358	0.06384548	2.5710260	-0.6095631	3.24443467	0.7653
		Magnesium	-2.5576545	-4.71447541	-0.4008335	-5.1641296	0.04882072	**0.0159**
		Superoxide dismutase	-2.9531672	-4.40721337	-1.4991211	-5.0161957	-0.89013876	**0.0013**
Kale	Photosynthetic constituents	Chlorophyll	3.8863578	1.9691767	5.803539	1.455842	6.316873	**<.0001**
		Instaneous photosynthetic rates	0.4155595	-0.5085015	1.339621	-1.341060	2.172179	**0.0014**
Spinach	Photosynthetic constituents	Carboxylation rate	-4.2918557	-8.3730841	-0.2106273	-11.488196	2.904485	**0.0393**
		Electron transport rate	0.0712477	-5.9023498	6.0448452	-8.343903	8.486398	0.2372
		Instaneous photosynthetic rates	-0.1115222	-6.0844583	5.8614140	-8.526203	8.303159	0.2574
		Photosynthesis	4.5287120	-1.5410242	10.5984482	-3.954954	13.012378	**0.0181**

		Photosynthetic rate	4.6194372	0.8019745	8.4368999	-2.430663	11.669538	**<.0001**
		SPAD	-1.6846919	-5.9011285	2.5317447	-8.958565	5.589181	**0.0006**
		Stomata conductance	4.5057552	0.6978593	8.3136510	-2.539170	11.550680	**<.0001**
Kale	Vitamin contents	Vitamin B	-0.65246636	-1.2294947	-0.07543803	-1.2294947	-0.07543802	**0.0267**
		Vitamin C	-0.09007755	-0.6228598	0.44270466	-0.6228598	0.44270466	0.1605
		Vitamin K	0.24986247	-0.2852683	0.78499330	-0.2852683	0.78499330	**0.0246**
Spinach	Vitamin contents	Ascorbate	3.0162682	2.0266557	4.005881	2.0247175	4.007819	**<.0001**
		Ascorbic acid	0.6759239	-0.6616565	2.013504	-0.6630911	2.014939	**0.0058**
Kale	Other phytochemicals	Aliphatic contents	-1.0087018	-1.9148506	-0.102553	-2.616960109	0.5995565	**0.0291**
		Crude fat	4.5217273	1.3674219	7.676033	1.099004426	7.9444502	**0.0010**
		Glucoalyssin	1.6428513	0.6739854	2.611717	-0.001560504	3.2872632	**<.0001**
		Glucoerucin	2.5298221	0.1946137	4.865031	-0.156920829	5.2165651	**0.0056**
		Glucoiberin	0.2262742	-1.6232679	2.075816	-2.051047718	2.5035961	0.2399
		Gluconapin	1.4310835	-0.5838671	3.446034	-0.982506202	3.8446732	**0.0304**
		Glucoraphanin	2.7185107	0.3163833	5.120638	-0.026594971	5.4636163	**0.0044**
		Indolyl contents	0.3972508	-1.4614943	2.255996	-1.887551716	2.6820534	0.1827
		Moisture & volatiles	-0.2630384	-2.1141366	1.588060	-2.541624284	2.0155475	0.4783

Spinach	Other phytochemicals	Ferric reducing-antioxidant power	0.6114968	-0.3389481	1.5619418	-0.3389481	1.5619418	0.2073
		Flavonoid	-0.5697040	-1.8899033	0.7504952	-1.8899033	0.7504952	0.1547
		Malondialdehyde	-4.9359522	-8.0081448	-1.8637597	-8.0081448	-1.8637597	**0.0007**
		Oxalic acid	-5.7037063	-8.4427129	-2.9646998	-8.4427129	-2.9646998	**<.0001**

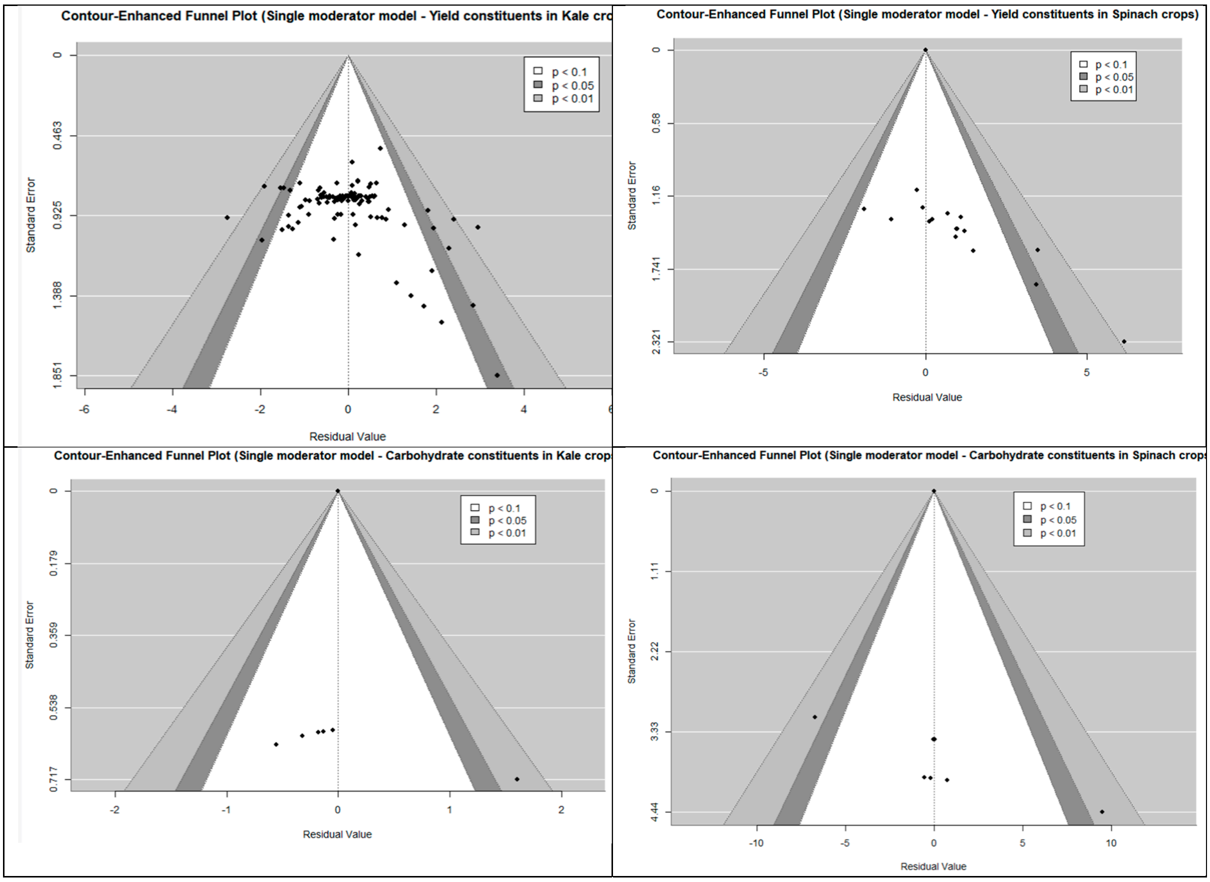

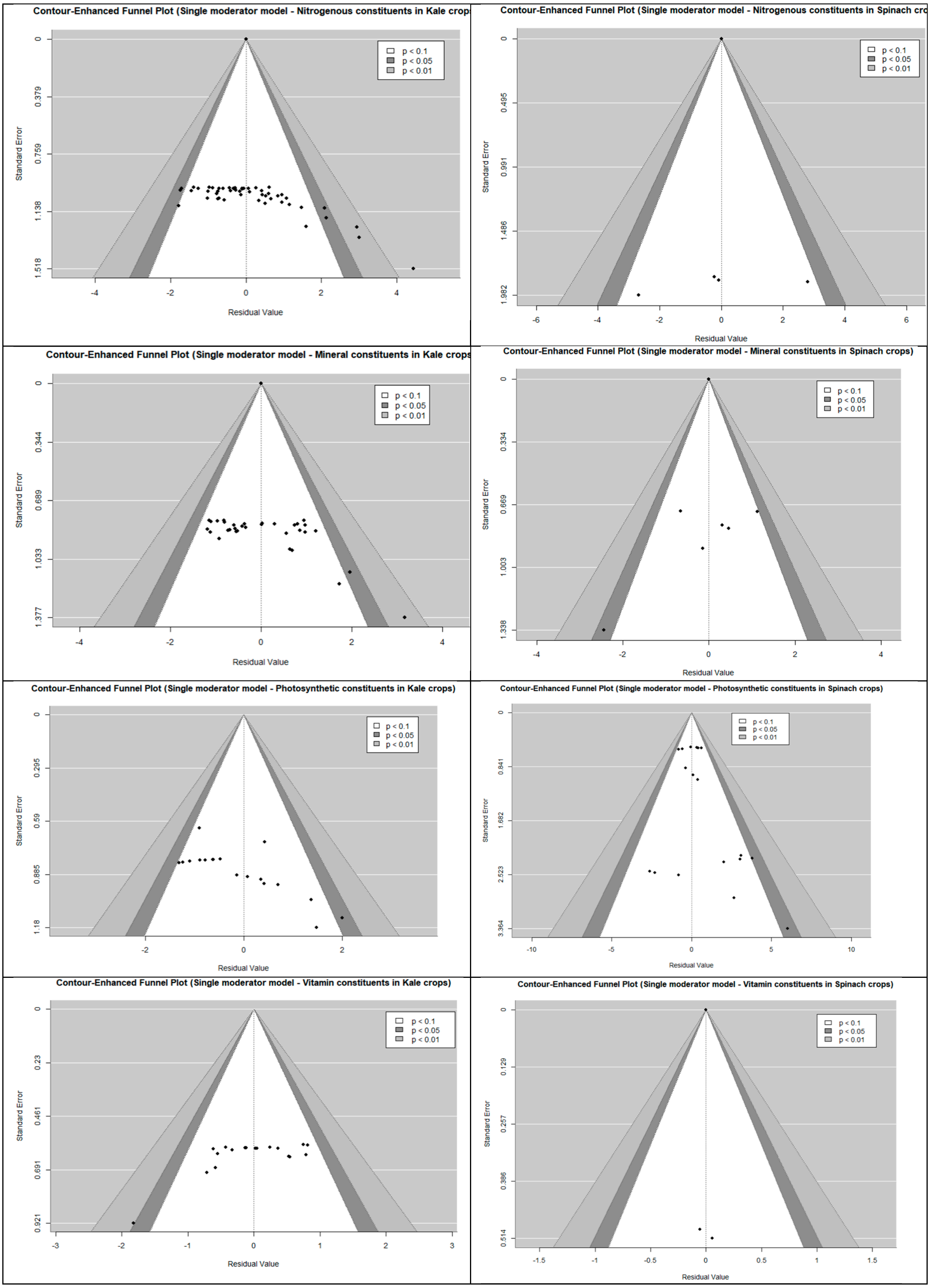

Contour-Enhanced Funnel Plot (Single moderator model - Nitrogenous constituents in Kale crop
Contour-Enhanced Funnel Plot (Single moderator model - Nitrogenous constituents in Spinach cro
Contour-Enhanced Funnel Plot (Single moderator model - Mineral constituents in Kale crops
Contour-Enhanced Funnel Plot (Single moderator model - Mineral constituents in Spinach crops)
Contour-Enhanced Funnel Plot (Single moderator model - Photosynthetic constituents in Kale crops)
Contour-Enhanced Funnel Plot (Single moderator model - Photosynthetic constituents in Spinach crops)
Contour-Enhanced Funnel Plot (Single moderator model - Vitamin constituents in Kale crops)
Contour-Enhanced Funnel Plot (Single moderator model - Vitamin constituents in Spinach crops)
p < 0.1
p < 0.05
p < 0.01
Standard Error
Residual Value

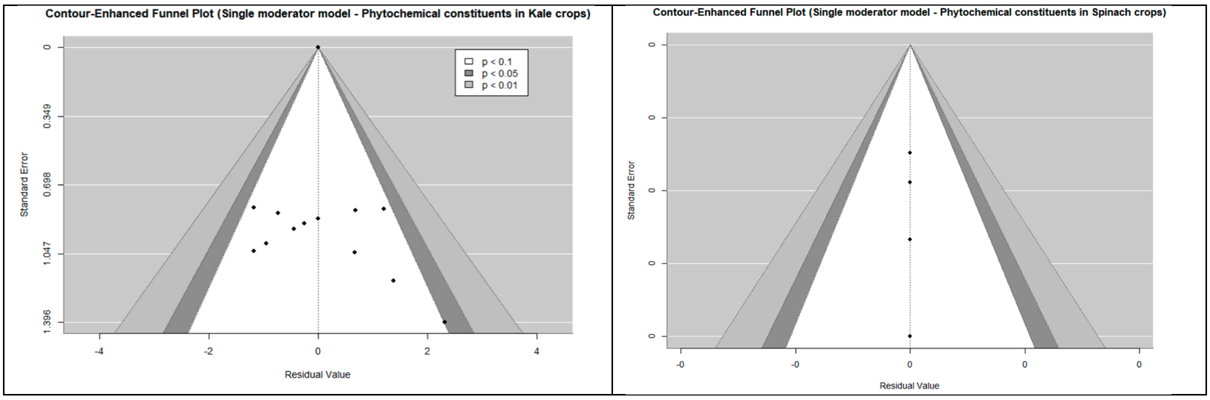

Supplementary Figure S14: Summary of funnel plots showing single-moderator model of the effects of eCO_2 on the different type of specific constituent measured in both kale (left-hand) and spinach (right-hand) crops.

Crop type by constituent group multi-moderator model

Here, two moderators were used to visualise the effect of eCO_2 across outcome categories by crop type (group * common-name)

Supplementary Table S10: Summary of the effect of eCO_2 across the different types of constituents measured in kale and spinach. Significant effect sizes are shown in bold.

Moderator level - Outcome category (group)	Moderator level - Crop type	Effect size estimate	Lower CI	Upper CI	Lower PR	Upper PR	p-value
Carbohydrate contents	Kale	0.97475038	-0.28291609	2.23241685	-2.207077	4.156578	0.1287
	Spinach	1.91624059	0.63801795	3.19446323	-1.273768	5.106249	0.2982
Mineral contents	Kale	0.67839349	-0.25059148	1.60737846	-2.388415	3.745202	0.5849
	Spinach	-0.87726861	-2.16600968	0.41147246	-4.071506	2.316969	**0.0041**
Nitrogenous compounds	Kale	1.38484020	0.54552515	2.22415524	-1.656007	4.425687	0.4602
	Spinach	0.08718589	-1.16838011	1.34275188	-3.093812	3.268184	**0.0114**
Other phytochemicals	Kale	1.02888428	0.01914237	2.03862620	-2.063345	4.121113	0.9271
	Spinach	-1.63586828	-3.33170454	0.05996798	-5.014945	1.743209	**0.0007**
Photosynthetic	Kale	1.03327409	0.10227630	1.96427188	-2.034145	4.100693	0.9226
	Spinach	1.41011047	0.35912564	2.46109531	-1.695831	4.516052	0.5167
Vitamin contents	Kale	0.30980987	-0.70890857	1.32852831	-2.785362	3.404982	0.2532
	Spinach	2.21393905	0.43577632	3.99210178	-1.207196	5.635074	0.3714
Yield	Kale	1.06228311	0.29399318	1.83057304	-1.959731	4.084297	0.8703
	Spinach	2.03161605	0.94960159	3.11363050	-1.084962	5.148194	0.9715

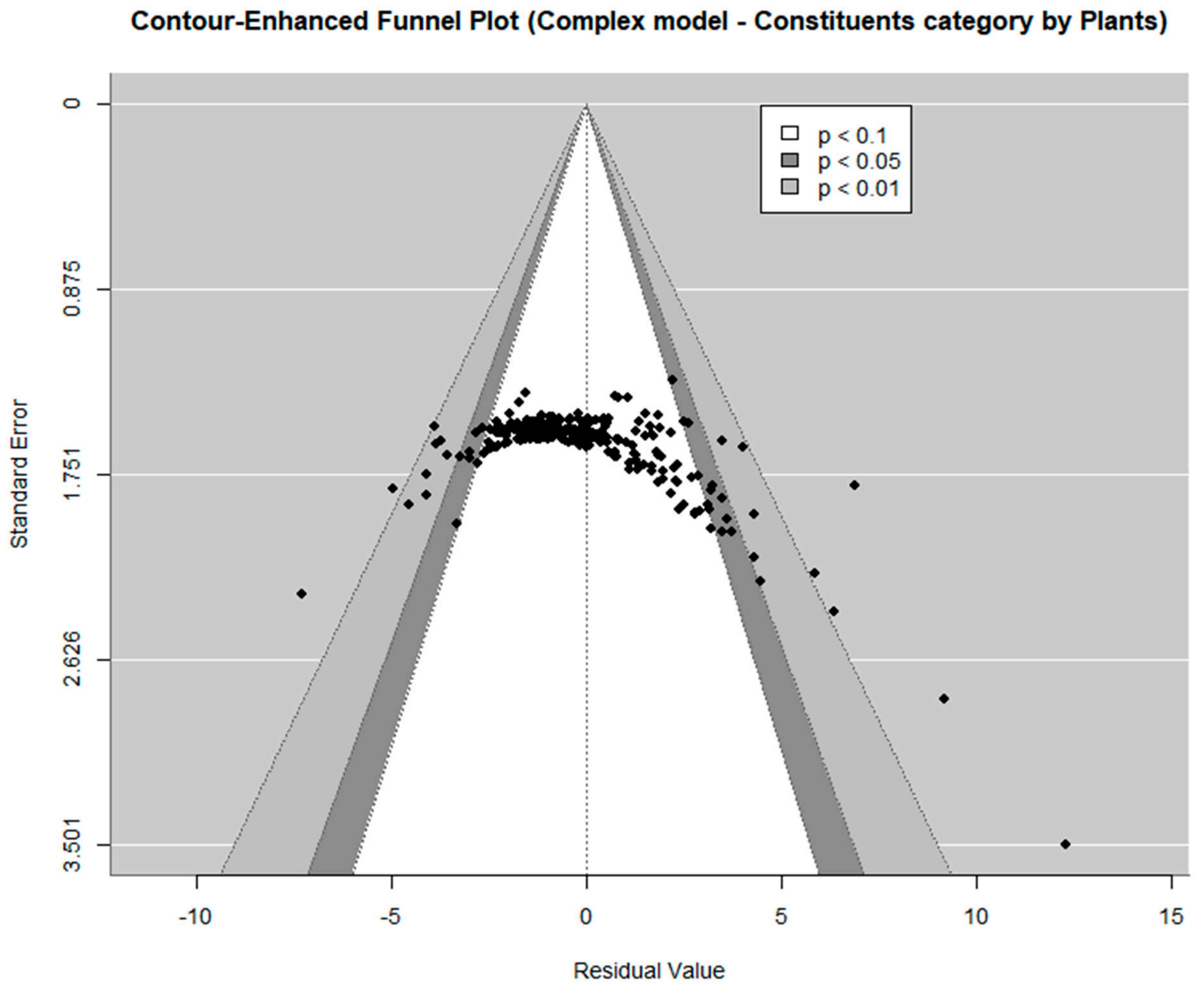

Supplementary Figure S15: Funnel plot for the complex multi-moderator model for the effects of eCO_2 on both kale and spinach within outcome categories. There is no evidence of asymmetry and, therefore, no sign of publication bias.
